# Unlocking the soundscape of coral reefs with artificial intelligence: pretrained networks and unsupervised learning win out

**DOI:** 10.1371/journal.pcbi.1013029

**Published:** 2025-04-28

**Authors:** Ben Williams, Santiago M. Balvanera, Sarab S. Sethi, Timothy A.C. Lamont, Jamaluddin Jompa, Mochyudho Prasetya, Laura Richardson, Lucille Chapuis, Emma Weschke, Andrew Hoey, Ricardo Beldade, Suzanne C. Mills, Anne Haguenauer, Frederic Zuberer, Stephen D. Simpson, David Curnick, Kate E. Jones

**Affiliations:** 1 Centre for Biodiversity and Environment Research, Department of Genetics, Evolution and Environment, University College London, London, United Kingdom; 2 Zoological Society of London, Regents Park, London, United Kingdom; 3 Department of Life Sciences, Imperial College London, London, United Kingdom; 4 Lancaster Environment Centre, Lancaster University, Lancaster, United Kingdom; 5 Graduate School, Universitas Hasanuddin, Makassar, Indonesia; 6 MARS Sustainable Solutions, Makassar, Indonesia; 7 School of Ocean Sciences, Bangor University, Askew Street, Menai Bridge, Anglesey, United Kingdom; 8 School of Biological Sciences, University of Bristol, Bristol, United Kingdom; 9 Australian Research Council Centre of Excellence for Coral Reef Studies, James Cook University, Townsville, Queensland, Australia; 10 Estación Costera de Investigaciones Marinas, Millennium Nucleus for Ecology and Conservation of Temperate Mesophotic Reef Ecosystems, Facultad de Ciencias Biológicas, Pontificia Universidad Católica de Chile, Santiago, Chile; 11 CRIOBE, PSL Research University, Moorea, French Polynesia; 12 Laboratoire d’Excellence “CORAIL”, Perpignan, France; University of Virginia, UNITED STATES OF AMERICA

## Abstract

Passive acoustic monitoring can offer insights into the state of coral reef ecosystems at low-costs and over extended temporal periods. Comparison of whole soundscape properties can rapidly deliver broad insights from acoustic data, in contrast to detailed but time-consuming analysis of individual bioacoustic events. However, a lack of effective automated analysis for whole soundscape data has impeded progress in this field. Here, we show that machine learning (ML) can be used to unlock greater insights from reef soundscapes. We showcase this on a diverse set of tasks using three biogeographically independent datasets, each containing fish community (high or low), coral cover (high or low) or depth zone (shallow or mesophotic) classes. We show supervised learning can be used to train models that can identify ecological classes and individual sites from whole soundscapes. However, we report unsupervised clustering achieves this whilst providing a more detailed understanding of ecological and site groupings within soundscape data. We also compare three different approaches for extracting feature embeddings from soundscape recordings for input into ML algorithms: acoustic indices commonly used by soundscape ecologists, a pretrained convolutional neural network (P-CNN) trained on 5.2 million hrs of YouTube audio, and CNN’s which were trained on each individual task (T-CNN). Although the T-CNN performs marginally better across tasks, we reveal that the P-CNN offers a powerful tool for generating insights from marine soundscape data as it requires orders of magnitude less computational resources whilst achieving near comparable performance to the T-CNN, with significant performance improvements over the acoustic indices. Our findings have implications for soundscape ecology in any habitat.

## Summary

Artificial intelligence has the potential to revolutionise bioacoustic monitoring of coral reefs. So far, only a limited set of work has used machine learning to train detectors for specific sounds such as individual fish species. However, building detectors is a time-consuming process that involves manually annotating large amounts of audio, followed by complicated model training. This process must then be repeated for any new dataset. Instead, we explore machine learning techniques for whole soundscape analysis, comparing the acoustic properties of raw recordings from the entire habitat. We identify multiple machine learning methods for whole soundscape analysis and rigorously test these using datasets from Indonesia, Australia and French Polynesia. Our findings reveal that use of a neural network pretrained on 5.2 million hours of unrelated YouTube audio offers a powerful tool to produce compressed representations of reef audio data, conserving the data’s key properties whilst being executable on a standard personal laptop. These representations can then be used to explore patterns in reef soundscapes using unsupervised machine learning, which is effective at grouping similar recordings together. We show these groupings hold relationships with ground truth ecological data, including coral cover, the fish community and depth.

## 1. Introduction

Effective monitoring of coral reefs is essential for supporting their conservation and restoration [1]. Monitoring data is typically collected using diver-led underwater visual census surveys. However, these diver-led surveys incur high expertise, logistical and financial costs [1,2]. Passive acoustic monitoring (PAM) presents an alternative means of gathering monitoring data which can be collected with greater ease and over extended periods [3,4]. However, in contrast to domains where PAM is well established (e.g., Northern European avian taxa), the species identity of any given sound in reef PAM recordings is usually unknown [5,6]. Instead, coral reef PAM often attempts to find relationships between the ecological community and the soundscape, the full extent of environmental sounds present [7]. Information held within reef soundscapes has been found to correlate with ecological community metrics (e.g., fish diversity, coral cover) [8–10] and can more readily capture temporal trends or the presence of cryptic organisms when compared visual survey methods [11–14]. However, PAM recording stations are typically fixed and the inter vs intra-site variability of the soundscape across these, as well as its relationships with key ecological processes, is poorly understood.

Given the ease of PAM recorder deployment and collection along biodiverse tropical coastlines, it is increasingly possible to collect datasets that scale well beyond the feasibility of manual analysis (e.g., months or years of raw audio) [2]. Automated analysis is therefore required to maximize the potential of these data. Previous attempts at automated analysis of whole soundscape data have primarily used acoustic indices. These indices are calculated by splitting long acoustic datasets into shorter samples (e.g., 5 sec) and using formulas that quantify one or more properties of each sample’s spectrogram or waveform (e.g., total amplitude, entropy across time) [15]. Comparisons of individual acoustic indices across groups or gradients, such as habitat types or levels of degradation, is common practice in the literature [16]. But indices typically share weak relationships with real-world ecological phenomena and are easily biased by non-target sounds such as anthrophony and geophony meaning the insights they generate are weak [15,17]. This is further compounded by a lack of standardisation for the many parameters required when calculating indices [17–19].

Machine learning (ML) represents a powerful alternative to the use of individual acoustic indices. ML algorithms can instead consider multiple acoustic indices in unison through their input as a multivariate feature vector into shallow ML algorithms (e.g., random forests, k-means clustering). In the case of soundscape ecology, these multivariate feature vectors are often referred to as a “compound index” [18]. Shallow ML algorithms can learn attributes of the data that integrate information across a compound index, uncovering emergent patterns or relationships that cannot be achieved with individual acoustic indices, better enabling them to perform tasks such as grouping or classifying new and existing data [15,20].

Given indices are designed by soundscape ecologists, these are typically considered “hand-crafted features”. Deep learning (DL), a subfield of ML, represents an alternative approach where the features are instead learned using deep neural network architectures which attempt to autonomously identify the most useful features from the data. These networks achieve this through using multiple layers of connected neurons, each performing a non-linear transformation to the input data. This allows networks to capture complex interactions within the data through an iterative learning process that optimises the values of these neurons for the task at hand [21]. Similarly to hand-crafted feature vectors, trained DL models can be used to output feature embeddings from samples which can be used as inputs to shallow algorithms, or the architecture can be adapted to output predictions directly. Deep-learned feature embeddings typically provide an improved performance over hand-crafted feature vectors at downstream tasks [21]. A downside of DL feature embeddings is that it is not easy to determine which aspects of the data relate to which features, meaning they offer a less interpretable “black-box” approach [22]. DL also requires significantly more computational resources as well as expertise in designing the training protocol and deploying this on accelerator chips [23].

Leveraging foundational pretrained networks is a powerful alternative to training DL models from scratch. These foundational models are typically trained on vast datasets to predict a broad selection of classes [24]. If the final classification layers are removed, these networks can be used as embedding extractors that generalize well to new tasks in a process known as “transfer learning” [25]. This process is typically orders of magnitude computationally cheaper, as each data point is only required to make one single forward pass through the network when compared to the dozens or more forward and backward passes required during the training process, however, the features remain a black-box.

Once multivariate feature representations of each data point have been obtained, these can be used as inputs for two common families of ML algorithms: supervised and unsupervised learning algorithms, though in the case of DL this can be achieved in a single integrated pipeline where features are learned in response to feedback from the algorithm. In soundscape ecology supervised learning usually involves training ML algorithms with labelled recordings, where labels correspond to a specific category in the case of classification tasks (e.g., habitat type) [20], or a value in the case of regression tasks (e.g., a biodiversity metric) [26]. The trained algorithm can then be used to recognise and predict these classes or values in new unseen data. Alternatively, unsupervised learning can be used which does not require labelled data, meaning it cannot directly predict classes or values, but instead can be used to identify patterns and structures within the data. In soundscape ecology, unsupervised learning can be used to identify groups of similar recordings using clustering algorithms (e.g., to identify similar habitats) [27], pseudo-label new recordings through semi-supervised learning [28], find anomalous events or periods (e.g., anthropogenic activity) [29], and more.

Due to its ability to provide improved analytical insights, ML is emerging as a powerful tool for automating the analysis of PAM data across multiple ecosystems and taxa. However, so far applications of ML to PAM data from natural habitats have primarily focused on generating detectors for specific taxa [24,25]. In the present study, we consider applications of ML to soundscape ecology, which uses the raw soundscape as a whole, rather than the individual acoustic events within [7]. Applications of ML in soundscape ecology represents an immature field, especially for coral reef habitats [21–23,30]. The optimum approaches to employ and limits to the insights these can unlock therefore remain unknown.

**Fig 1:**
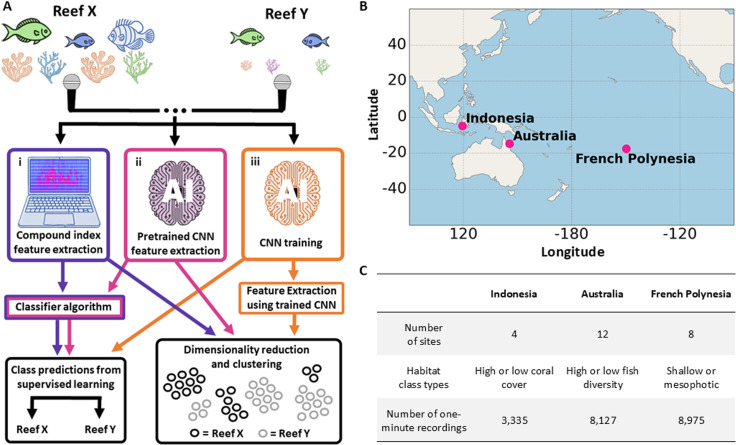
(A) Analytical workflow used on each coral reef soundscape dataset. Classification and clustering algorithms were trained on features extracted using either the compound index, pretrained CNN or trained CNN to identify habitat and site classes. For the trained CNN, classification was performed directly using the trained network. (B) Geographic location of the three datasets. (C) Table with key information from each dataset. Images were created using DALL-E 3 and the shapes tab in Microsoft PowerPoint. The map was created using public domain Natural Earth data (https://www.naturalearthdata.com).

To better understand the potential of ML to support coral reef soundscape ecology, we compared different feature extraction methods and downstream algorithms at a range of tasks. We compared the efficacy of the three methods for extracting features representations from coral reef soundscape data: a compound index, a pretrained foundational convolutional neural network model (P-CNN) trained on 5.2M hrs of unrelated YouTube audio (VGGish) [31], and the same network custom trained on reef soundscape data (T-CNN) (Fig 1A). We performed this comparison through assessing their performance at supervised and unsupervised ML tasks. To ensure that our results were representative of reef soundscapes broadly, we used recordings from three biogeographically distinct locations (Fig 1B) [32]. Using supporting meta-data available with each dataset, we split each into two unique habitat types: high or low coral cover for Indonesia, high or low fish diversity for Australia, and shallow or mesophotic depths for French Polynesia (Fig 1C). We tested the ability of the ML algorithms to identify these habitat groupings from three distinct coral reef soundscape datasets, and then tested their ability to identify individual sites within each dataset regardless of habitat grouping. The three datasets were treated independently throughout to provide a suite of unique challenges. We report on both the performance of each feature representation as well as the strengths and weaknesses of unsupervised vs supervised learning at these tasks.

## 2.0 Methods

### 2.1 Datasets

For this study, we pooled three tropical coral reef PAM datasets, each with different supporting ecological or geographical data. These were collected from three distinct coral reef biogeographic realms: i) South Sulawesi, Indonesia, in the Tropical Indo-Pacific; ii) Lizard Island, Australia, in the Coral Sea; and iii) French Polynesia, in the Mid-South Tropical Pacific (Figs 1B and S1) [32]. Recording periods from the Australian and French Polynesian sites were sub-sampled to reduce dataset size (S1 Text). From each dataset, the supporting metadata enabled the division of recording sites into two classes. These classes represented divisions between classes for which the literature suggests there is strong evidence that differences in the soundscape should be expected [5,9,33]. This strategy enabled us to devise tasks with which we could compare the different ML methods in question, but we do not test more challenging differences or gradients at this stage. For the Indonesian data, sites were divided into high (91.2-93.1%) and low (2.1-17.6%) coral cover groups. The Australian sites were divided into two classes, one with high biomass (20.5-52.2 kg) and species richness (45spp - 53spp) scores per transect, and another with low biomass (5.2-9.8 kg) and species richness (30spp – 35spp) scores per transect (S2 Fig), which we refer to as high- and low-fish-diversity sites from here on. The French Polynesian sites were divided into shallow (10-15m) and mesophotic (55-65m) depth classes.

Each dataset was collected using SoundTrap hydrophone recorders (SoundTrap 300ST, Ocean Instruments, Auckland, NZ) which were calibrated by the manufacturer with a flat frequency response. These were suspended 0.5 m above the seabed using a sub-surface buoy. To mitigate against instrument bias, the individual recorders were frequently rotated between the different respective sites within the Indonesian and Australian datasets, however, logistical constraints of deploying recorders at mesophotic depths prevented this for the French Polynesia dataset. The full sampling regime and recorder rotation is detailed in S1 Text. To minimise the introduction of geophonic noise, all recordings were taken during seastates between 0–2 on the Beaufort scale and never during periods forecasted to rain, where conditions deviated from this, recordings were not taken. All recordings were taken in remote areas away from frequently trafficked boating channels and mooring points to minimize the presence of anthropogenic noise, a check for boat noise was also performed to ensure its prevalence was low (S2 Text). In total, 3335, 8127 and 8925 one-minute recordings were used from the Indonesian, Australian and French Polynesian datasets respectively. Further details including, maps, recording schedules, and instrument rotations, are available in S1 Text and S1 Fig.

### 2.2 Extracting feature embeddings

Each dataset was divided into one-minute recording periods and embeddings were extracted from these. Guidance from previous literature was used to assemble a compound index (Table 1) [15,20,26]. The aim was to include a broad selection of features, whilst preventing this from including unnecessary noise which can reduce the performance of machine learning algorithms. This began with the identification of suitable acoustic indices using the following criteria: (i) previous use in the literature on marine soundscape recordings, (ii) do not require recorder calibration, which would preclude the use of more widely accessible recorders [34]; (iii) could be computed using existing toolkits in a single programming language (e.g., Python). Of the identified indices, the acoustic diversity index (ADI) and acoustic evenness (AEI) have previously been reported to strongly covary [20]. Therefore, to reduce the introduction of noise only AEI was used (Table 1). Seven of the eight indices were then calculated across three different frequency bands: a low-frequency (0.05–2 kHz) where fish sounds dominate [5], a medium-frequency band (2–8 kHz) where snapping shrimp sound dominates [20] and a full band (spanning 0.05–8 kHz). We excluded frequencies below 0.05 kHz from the low- and broad-frequency band recordings to remove geophonic noise and self-noise from the recording system [35]. The exception was the normalised difference soundscape index (NDSI), which requires the input of two bands, for which 0.05–1 kHz and 2–5 kHz bands were used as in Williams et al., (2022) [20]. Recordings were split into 0.96-second segments (to match the CNNs input windows), totalling 62 per minute, and indices were calculated for each segment. The mean and standard deviation of these were then taken for each minute [15], providing a 44-dimension embedding which was used as the compound index to represent each one-minute recording. All processing was performed in Python (v3.7) with the scikit-maad package (v1.3) [36].

**Table 1:** Acoustic indices used in the compound index. Indices were calculated using the Scikit-maad (v1.3) package. Where additional settings are ‘None’, the index was calculated over the precomputed spectrogram with no further parameters required. The reference column cites a study which has reported a relationship between the respective index and at least one aspect of coral reef ecology.

Index	Mechanism	Additional settings	Reference
Acoustic Complexity Index (ACI)	Measures variability in intensity of frequencies across time	None	Bertucci et al., (2016) [33]
Acoustic Diversity Index (ADI)	Measures diversity across frequency bands	Min and max frequencies matched the frequency band in use. bin_step was 1/10th of the bands range. dB threshold = -50.	Williams et al,. (2022) [20]
Acoustic Entropy (H)	Measures randomness across temporal and spectral domains	s = QUT, mode = fast, Nt = 256	Bertucci et al., (2016) [33]
Amplitude Index (M)	Measures median of amplitude envelope	mode = fast, Nt = 256	Williams et al,. (2022) [20]
Bioacoustic Index (BI)	Measures cumulative intensity across frequency bands	Min and max frequencies matched the frequency band in use	Elise et al,. (2019) [37]
Normalised mean difference index (NDSI)	Measures amplitude difference between two selected frequency bands	flim_bioph = (2000, 8000), flim_antroPh = (50, 2000)	Elise et al,. (2022) [4]
Spectral entropy (Hf)	Measures randomness across the frequency domain	None	Elise et al,. (2019) [37]
Temporal Entropy (Ht)	Measures randomness across the temporal domain	mode = fast, Nt = 256	Elise et al,. (2019) [37]

For the pre-trained convolutional neural network (P-CNN), we selected VGGish due to its successful application in previous work on terrestrial soundscape data [29,38]. VGGish was trained on the YouTube-100M dataset, a diverse collection of YouTube audio clips totalling 5.2 million hours in length, to produce a highly generalisable audio embedding extractor [31]. It uses a version of the Visual Geometry Group object recognition architecture that was adapted for audio input [39]. The P-CNN pre-processing down-samples recordings to 16 kHz and divides them into non-overlapping 0.96 second audio frames, processed through a Short-time Fourier Transform with 25 ms windows every 10 ms. These are integrated into 64 mel-spaced frequency bins which are log-transformed to produce a 96 × 64 bin log-mel spectrogram which matches the network’s input shape. Further details are available in Hershey et al., (2017) [31]. This P-CNN can be configured to output the 128-dimension feature set from the penultimate layer in place of the classification head. We averaged these feature values from each one-minute recording to produce a single embedding. Processing was performed in Python (v3.7).

To ensure comparability with the P-CNN, for the trained CNN (T-CNN) we trained VGGish on each respective reef soundscape dataset and task from scratch on an NVIDIA A100 GPU. The pre-processing protocol used for the P-CNN was replicated to produce 960 ms log-mel spectrogram samples. The order of these samples was shuffled and minibatches of 32 samples were used during training. The number of output nodes of the network was adjusted to match the number of target classes in each task. The T-CNN was then trained for 50 epochs, with inference on the validation data every epoch. The epoch which reported the highest validation accuracy was then used for inference on the test data. All other hyperparameters followed the default settings for VGGish used in Hershey et al (2017) [31], including randomly initialized weights using a standard deviation of 0.01, a learning rate of 0.001, and an Adam optimiser. Other parameters specific to unsupervised and supervised learning are outlined in sections 2.3 and 2.4 respectively, all other parameters can be found in the vggish_params.py script (see the Code reporting section). Processing was performed using Tensorflow (v1.15) and scikit-learn (v0.22) in Python (v3.7).

### 2.3 Unsupervised clustering

Unsupervised clustering was used to reveal structures and patterns in the data without relying on pre-labelled classes. Embeddings from the compound index and P-CNN were extracted from recordings. To generate embeddings using the T-CNNs, the VGGish CNN was trained for 50 epochs on each dataset to predict which site recordings originated from within the respective dataset (see section 2.4 for more detail). The final layer of the trained models were then removed to produce three networks comparable to the P-CNN, except these were now trained on reef soundscape recordings from the three respective datasets. These T-CNNs were then used as pretrained embedding extractors on all recordings from their respective datasets in the same manner as the P-CNN. Importantly, only the T-CNN’s trained on the site classes, not habitat classes, were used for embedding extraction in unsupervised learning. This was to avoid introducing ecological context into the model training, which may not be available in exploratory analyses this experiment was simulating, whereas the site of origin should typically be available.

To determine which of the three embedding extraction methods was most proficient at producing embeddings that represented known properties of the data, a qualitative assessment was first performed using UMAP visualisation in two dimensions with UMAP’s associated Python package (v0.5.3) [32]. For all six tasks performed by each method, a plot was produced where points were labelled with their known class, and the fidelity of groupings to their true class compared between each method.

A quantitative assessment was also undertaken using affinity propagation clustering [40,41]. This algorithm was selected as it does not require the number of clusters to be predefined, instead the affinity propagation algorithm identifies this, simulating analysis with an entirely unlabelled dataset where number of classes is unknown. To improve clustering, UMAP was used to reduce the embeddings of each dataset to 10 dimensions [42]. For a given method and task, the cluster to which each recording was assigned was entered into a contingency table against its known class. Models most proficient at clustering recordings from the same class, while excluding recordings from other classes, generated clusters with a higher fidelity to individual classes. Those less proficient assigned recordings more randomly across clusters. A chi-squared test was performed on contingency tables to assess this, where a higher score indicating models with a higher fidelity to true classes. Processing was performed using scikit-learn (v0.22) in Python (v3.7).

### 2.4 Supervised classifiers

All three ML methods were set two classification tasks, habitat type and individual site identification, from each of the three soundscape datasets. The habitat classification tasks were as follows: high or low coral cover sites for the Indonesian dataset, high- or low-fish-diversity sites for the Australian dataset, and shallow- or mesophotic-depth sites for the French Polynesian dataset. For each task, recordings were split into training, validation and test sets, where 66–75% of the data was used for training and the remaining data was evenly split into validation and test sets (S1 Text). To account for temporal autocorrelation in data and recorder bias, these divisions were carefully performed to exclude entire sites from the training data where possible, or extended contiguous recording periods if not, see S1 Text for full details on each dataset. The validation and test accuracies were carefully monitored to ensure clear over or underfitting did not occur, these values can be found in the cnn_predictions.zip within the supporting Zenodo repository (see Data Availability).

For the compound index and P-CNN embeddings, random forest ML classifiers were trained for each task. Random forests were selected due to their previous successful implementation in the soundscape literature [15,26], robustness to overfitting, and low computational costs, enabling computation on personal devices. For each task, fifty random forest classifiers were trained and then used for inference on the validation data. The instance which reported the highest validation accuracy was used for inference on the test data, and the accuracy of this reported. As classes were well balanced within each task, raw accuracy (the proportion of one-minute recordings correctly classified) was used as the performance metric. For each task, this process was repeated multiple times using carefully selected splits of the data into training, validation and test sets. To ensure the models were tested on novel data that was out-of-distribution from the training data, the data was grouped into blocks of entire sites and/or dates and splits were devised using these to hold out entire blocks for testing, rather than random assignment (S1 Text). To ensure the data splits selected did not bias any approach, one hundred repetitions were performed for each task, except for the Australian and French Polynesian habitat classification tasks, where only 32 were used as this was the maximum number of possible combinations which enabled the exclusion of entire sites from the training data (S1 Text). Analysis was performed using the default random forest parameters in scikit-learn (v0.22) with Python (v3.7).

For the T-CNNs, networks were trained on the reef soundscape dataset for each individual task. The most common class prediction across all 0.96 second segments in a one-minute recording was used as the class prediction for each minute in the test data. This process was repeated 100 times using the same training, validation and test combinations as the compound index and P-CNN. This workflow was performed using Tensorflow (v1.15) in Python (v3.7).

Analysis of variance (ANOVA) tests were used to determine whether significant differences existed between the accuracy of each embedding approach across all repetitions for each task. Where significant differences were detected (p < 0.05), post hoc Tukey tests were used to determine which methods differed; performed using the scipy library (v1.73) in Python (v3.7).

## 3. Results

### 3.1 Exploring coral reef soundscapes with unsupervised learning

**Fig 2:**
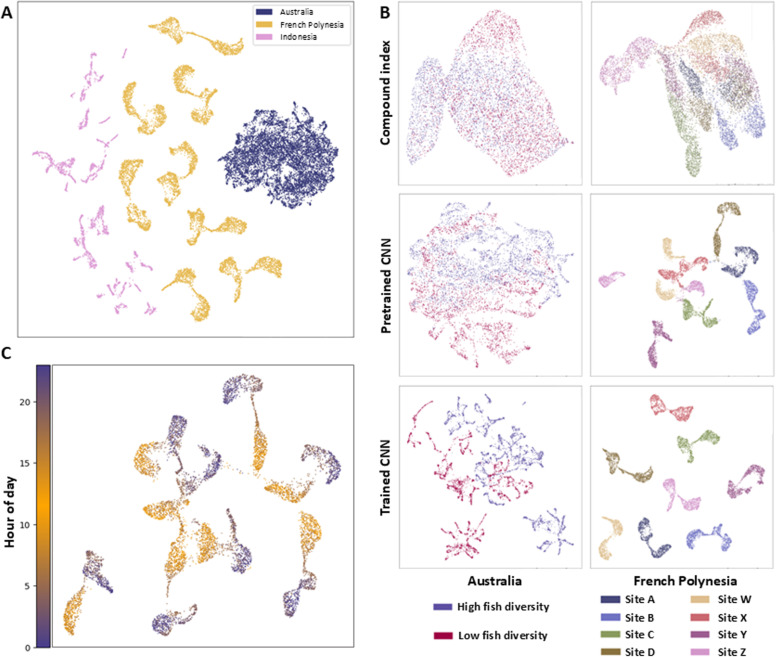
(A) Uniform manifold approximation (UMAP) dimensionality reduction visualisations of all three datasets produced using the pretrained CNN, individual points represent a one-minute recording. (B) Selected UMAP visualisations of the compound index, pretrained CNN and trained CNN embeddings. visualisations are labelled with colours corresponding to the habitat class (high or low fish diversity) or site. The remaining visualisations can be found in S3 Fig. (C) UMAP visualisation of the French Polynesian dataset which reveals temporal patterns within the dataset produced using the pretrained CNN. The timescale runs from 00:00am (hour of day = 0) to 23:59pm (hour of day = 24).

Uniform Manifold Approximation and Projection (UMAP) plots were used to quantitatively explore patterns in the soundscape. When interpreting UMAP plots, it is important to note that while UMAP accurately maps local similarities within the data, allowing it to identify clusters, the spatial arrangement of these clusters is less reliable [43]. A visualisation of the three full datasets (Indonesia, Australia, French Polynesia) using the P-CNN UMAP embeddings showed that each dataset formed distinct groups, separate from the others, indicating that the recordings from each location had unique properties (Fig 2A).

Qualitative inspection of UMAP visualisations from each individual dataset also revealed that the known habitat classes were frequently key drivers behind the clusters that were formed, indicating the influence of these on the soundscape (Figs 2B, and S3A,S3B and S3C) [29]. Of the three embedding methods, the recordings were most clearly separated into discrete clusters that conformed to habitat classes by the T-CNN, followed by the P-CNN (Figs 2B and S3). The UMAP plots also revealed a unique soundscape “fingerprint” for many sites, though this effect was strongest for the French Polynesian dataset where recording instruments could not be rotated between sites (Figs 2B and S3 S1 Text). In addition to identifying site and habitat classes, UMAP plots also revealed temporal patterns within the data (Fig 2C and S4-6;S3 Text). For example, crepuscular periods linked separate night and day clusters for the French Polynesian dataset (Fig 2C), while the soundscape of two sites in Indonesia converged around the new moon (4-S6 Figs; S3 Text).

Chi-square scores from affinity propagation clustering were highly significant *(p* < 0.001) for all three embedding types across each dataset (Table 2), indicating the clustering algorithm was able to non-randomly assign recorders to clusters that corresponded to habitat or site classes in every case. Overall, the T-CNNs yielded the highest chi-square scores, indicating greater fidelity to both habitat and site classes. In every instance, this was followed by the P-CNN and then compound index (Table 2). However, for the French Polynesian habitat and site identification tasks, the chi-square scores for the T-CNNs were only marginally higher than those of the P-CNN (Table 2). This was particularly notable for the site identification task, as the T-CNN embeddings were derived from networks explicitly trained for site classification, underscoring the strength of the P-CNN.

**Table 2:** Chi-squared scores (x 10^3^) generated from contingency tables of each recording’s true class and the cluster that each recording was assigned to by affinity propagation clustering. Higher scores indicate that clusters better represented true classes. All chi-square scores were highly significant (p < 0.001).

Dataset	Task	Compound index	Pretrained CNN	Trained CNN
Indonesia	Habitat: high or low coral cover	1.72	2.66	3.13
Indonesia	Site identification	6.05	8.58	9.53
Australia	Habitat: high or low fish diversity	1.58	3.46	5.06
Australia	Site identification	14.64	36.36	72.61
French Polynesia	Habitat: shallow or mesophotic	6.97	8.67	8.76
French Polynesia	Site identification	45.00	60.51	62.83

### 3.2 Predicting habitat class and site identity with supervised learning

Overall, the classifiers performed well in predicting habitat and individual site classes from one-minute reef soundscape recordings. The six tasks exhibited a range of difficulty, with a mean accuracy across all tasks ranging from 0.56 (±0.05) to 1.00 (±0.0) corresponding to a performance above random classification ranging from 0.23 (±0.11) to 0.87 (±0.0) (Table 3 and S7 Fig). The strongest performance at site classification tasks was reported for the French Polynesian dataset and the weakest was reported for the Australian dataset. However, we find the training and test divisions selected for a given repeat strongly influenced results. For example, the French Polynesian habitat classification task had a mean accuracy of 0.83 using the T-CNN across repeats, but the standard deviation was high (±0.2), with some repeats failing to improve beyond random classification.

**Table 3:** Mean and standard deviation of classifier accuracy across repeated training instances using each the three machine learning methods (compound index, pretrained CNN and trained CNN) at six different tasks. Accuracy is the proportion of one-minute recordings from the test data that were correctly classified. Methods where accuracy was reported as significantly higher by the ANOVA test are indicated in superscript next to the mean value for the respective method (A = highest group, B = second highest group, no letter = lowest group). The Random baseline accuracy indicates the expected accuracy of a model that performs random classification. N = 100 for all tasks, except the Fish diversity (Australia) and Depth (French Polynesia) tasks, where N = 32.

	Compound index		Pretrained CNN		Trained CNN		Random baseline accuracy
Task	Mean	Standard deviation	Mean	Standard deviation	Mean	Standard deviation	
Coral cover (Indonesia)	0.86	0.10	0.88	0.08	0.91^A^	0.09	0.50
Site identification (Indonesia)	0.85	0.09	0.85	0.08	0.89^A^	0.08	0.25
Fish diversity (Australia)	0.71	0.12	0.72	0.09	0.73	0.11	0.50
Site identification (Australia)	0.56^A^	0.05	0.52	0.07	0.54	0.09	0.08
Depth (French Polynesia)	0.88	0.16	0.82	0.20	0.83	0.20	0.25
Site identification (French Polynesia)	0.99^B^	0.00	0.99	0.00	1.00^A^	0.00	0.13

The three embedding approaches reported comparatively similar accuracies to one another (Table 3). ANOVA tests reported no significant difference between any of the three methods for the habitat identification tasks set from the Australian and French Polynesian datasets (Table 3). However, significant accuracy differences were observed between methods for the Indonesian habitat classifier and for site identification classifiers across datasets. For three of the four tasks where a difference was observed, the T-CNNs outperformed the other two methods, including the habitat and site classifiers for the Indonesian dataset and the site identity classifier for the French Polynesian dataset. However, the compound index had significantly greater accuracy than the P-CNN and T-CNN for the Australian site classifier.

Inspection of the confusion matrices used to interpret classifier performance revealed that the three embedding extraction methods reported similar patterns of misclassification for each task, where samples from any given class were assigned to the same incorrect classes across methods (S8A, S8B and S8C Fig). The only exception was the French Polynesian habitat classification task where the random forests trained on the compound index and P-CNN were more likely miss-classify shallow samples as mesophotic, whereas the T-CNN was more likely to misclassify mesophotic samples. The only instance where a class was misclassified the majority of the time across all repeats was for site A in the Australian dataset, where samples were frequently assigned to class G (S8B Fig). This misclassification was likely due to the close proximity of these sites (S1 Fig) and their habitat attributes, both of which were ‘low’ fish diversity sites.

### 3.3 Benchmarking against current automation: acoustic indices

**Fig 3:**
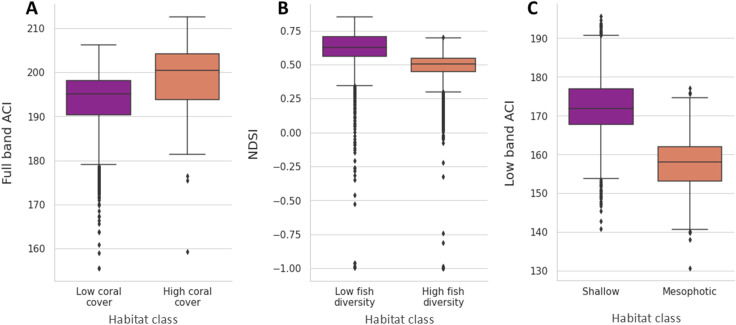
Boxplots of the acoustic index for which the highest significant difference between habitat classes was reported for the (A) Indonesian, (B) Australian and (C) French Polynesian datasets. Boxes and their bars represent the 25th, 50th and 75th quartiles.

Statistical comparisons of individual acoustic indices represent the predominant method for automated analysis of reef soundscape data [19,44,45]. To assess the performance of individual indices, we deliberately selected those that showed the most significant differences between habitat classes. These were the full-band acoustic complexity index (ACI), the normalised difference soundscape index (NDSI), and the low-band acoustic complexity index (ACI), respectively (Fig 3), which reported Mann-Whitney U test results of: U = 2.00 x 10^6^, p < 0.0001, U = 7.71 x 10^6^, p < 0.0001, and, U = 3.64 x 10^6^, p < 0.0001. Despite this biased selection, our analysis showed that these indices were still unable to classify individual recordings with the same accuracy as ML methods (Fig 3). The proportions of recordings that could be unambiguously classified as the correct habitat type were only 5.2%, 13.0%, and 12.2% for each dataset respectively. Even the lowest-performing ML classifiers, across all tasks and repeats, achieved accuracies of 58.1%, 57.7%, and 47.1% for each embedding type (Table 3). Furthermore, individual acoustic indices failed to classify the site of origin for the recordings (S9 Fig).

## 4. Discussion

### 4.1. Machine learning unlocks greater insights from coral reef soundscapes

Using three biogeographically independent datasets, we demonstrate the potential of machine learning (ML) to automate the large-scale analysis of coral reef soundscape data (20,437 one-minute recordings in the present study). Previous work using automated techniques has predominantly used individual acoustic indices, which can reveal relationships between the soundscape and ecological attributes on reefs [10,33,46–48]. However, comparing individual indices provides only a weak signal [2,28,49,50], which is supported by our findings (Figs 3 and S9). Instead, we show that combining features into embeddings and using these as inputs for ML algorithms provides a powerful alternative, especially when using embeddings output by deep-learning models. We find unsupervised learning algorithms can be used to effectively explore and understand trends within these data. However, to ensure the reliability of ML-based soundscape analysis, careful consideration must be given to sampling design and data partitioning to mitigate biases from temporal variability, instrumentation, and other influences [51].

Looking to the ecological properties of our datasets, these results further support the hypothesis that the raw soundscape of coral reefs shares a relationship with properties such as fish community composition and coral cover (Fig 2 and Table 3). This relationship likely reflects variations in the soniferous community, primarily fish and sound-producing invertebrates, which are known to correlate with coral cover [8,10,33], despite corals themselves not contributing directly to the soundscape. We also provide further evidence that shallow and mesophotic reefs often exhibit distinct soundscapes [45,52–55] (Fig 2 and Table 3). More research is needed to understand the factors that drive this difference, such as the ecological community or oceanographic conditions [56].

Our findings provide further evidence that temporal patterns influence reef soundscapes [11,57], with clusters typically grouping recordings from similar time periods (Figs 2C and S4-S6). For example, crepuscular periods linked separate night and day clusters for the French Polynesian dataset (Fig 2C). Furthermore, ML was often able to differentiate individual sites, an insight which could not have been elucidated using acoustic indices (Fig 3). However, our site identification findings underscore the need to mitigate recorder bias, as models may instead learn to identify properties of recordings from specific devices, a common issue in machine learning analyses. Notably, the French Polynesian dataset exhibited the most pronounced site differences, likely as it was the only dataset in which the instruments could not be rotated (S1 Text).

When comparing the performance across datasets, the unique biogeography, instrument rotation strategy, and sampling regime across time likely interact, making it difficult to disentangle which of these factors had the strongest impact on performance. Unsupervised learning further confirmed that each dataset exhibited unique properties (Fig 2A), likely due to both the soundscape properties and the artefacts of individual recording devices used, highlighting the need for caution when attempting to infer ecological variables using models or findings from another dataset [37].

### 4.2 Performance and comparison of machine learning approaches

Regarding the selection of the optimal feature extraction approach for ML, a clear difference in performance between the methods was revealed by the unsupervised learning tasks. Here, the T-CNNs produced clusters with a notably greater fidelity to true classes for every task compared to the compound index and in most tasks when compared to the P-CNN (Fig 2C and Table 2), which in turn outperformed the compound index. These results reveal that feature embeddings obtained from deep learning yield improved outputs compared to handcrafted compound index embeddings. Notably, this represents the first demonstration that transfer learning using a network pretrained on data from an entirely different audio domain, with the 5.2m hrs of YouTube audio used to train VGGish, can be successfully applied to coral reef soundscapes. Further improvements to feature embeddings may be achieved by testing alternative DL embeddings developed on large bioacoustic datasets and by incorporating temporal information or architectures that capture long-range dependencies, such as transformers [58,59].

When considering the computational costs, generating feature embeddings with the T-CNN was significantly more computationally intensive than the other two methods. The Indonesian dataset represented the smallest of the three, with 3,335 one-minute recordings. Training each repeat of the T-CNN site classifier on the Indonesian dataset took approximately one-hour to train on an NVIDIA A100, an often prohibitively expensive piece of equipment to access. Training with a CPU available on a standard personal computing device was unable to complete even one of the 50 training epochs in a 24 hour period. Conversely, the computational resources required to run the compound index and P-CNN are much more accessible, taking 84 and 96 minutes respectively to extract embeddings from all recordings in the Indonesian dataset. Following embedding extraction, the execution time of downstream processing using random forest classifiers and unsupervised learning was negligible on a CPU.

When selecting an unsupervised or supervised approach, we find that unsupervised learning offers several advantages over supervised learning. Firstly, the natural overlap that can occur between soundscapes across time is more gracefully reported by unsupervised learning which considers the full dataset through cluster overlap or visualisations (Table 2 and Fig 2), whereas this often leads to a classification error if using supervised learning (Table 3). Additionally, various measures can be derived from unsupervised outputs, such as cluster diversity across recording samples to identify more variable soundscapes. Additionally, percentage overlap in cluster allocations and centroid distances within the embedding space can be used to assess soundscape similarity between sites [29,60]. Prior knowledge on the ecological attributes of certain sites could also be used in a semi-supervised way to infer these from other sites [61].

For supervised classification tasks, our findings show that the difference in performance between the three embedding extraction methods on reef soundscape data was minimal. Multiple algorithms and their respective hyperparameters are available. However, it is well documented that as dataset size grows the choice of algorithm matters less [62]. Our findings support this in the context of the large soundscape datasets used here, given the broadly similar accuracy scores across embedding types (Table 3). Furthermore, raw soundscape datasets of this scale are easily gathered, meaning more computationally intensive embedding types have diminishing returns.

Moving forward, for guaranteed optimal performance, we recommend developing T-CNNs specific to each dataset for use in unsupervised approaches, training them on known attributes of the data (e.g., site classes, as used here). However, the P-CNN offers near comparable performance for orders of magnitude less computational cost. The P-CNN also outperforms the compound index and represents a more standardised tool than this index, which requires curation from an extensive set of indices and parameters that currently vary from study to study [44,49]. The standardised features of a P-CNN facilitate easier comparisons across datasets, whereas a T-CNN would require retraining. We therefore recommend a P-CNN for most use cases. Outputs from clustering can then be used in downstream statistical models, for example testing the relationship between ecological metrics and soundscape diversity of sites which can be indicated by the number of clusters each site is assigned to, or, comparing the overlap between clusters of sites where an intervention has been implemented (e.g., reef restoration) with healthy and degraded baselines (see the tutorial in the Code Reporting section).

### 4.3 Future directions

There are multiple ways future research could build on the findings from this study. Firstly, soundscape data alone can be used for rapid comparisons between different treatments in novel contexts (e.g., restoration, degradation) without requiring additional biodiversity metrics [20,59]. However, integrating standardised ecological survey data across diverse settings and biogeographies is essential for identifying the underlying drivers. Key areas for investigation include determining how reliably the soundscape can serve as an indicator of soniferous taxa, their relationship with broader ecosystem functioning, and how this changes over time [45,48,57]. Such data would also help address the black-box challenge by linking soundscape properties to ecological attributes. While the tasks examined in this study were achievable with our methods, these relationships and their limitations could be better explored by integrating multiple ecological measures and assessing them across gradients rather than through discrete binary classification challenges we devised. For example, future studies could use metrics such as fish diversity or coral cover from sites spanning biogeographical or human pressure gradients. Metrics with the strongest relationships to the soundscape could be identified and used to predict these on new sites using low-effort PAM data collection. Crucially, prospective users should follow best practices when assembling datasets, ensuring control over confounding variables that may affect machine learning algorithms, such as instrument bias, temporal autocorrelation, and careful curation of training and test sets [63–65].

The availability of low-cost recording technology [34,66] and rapid insightful analysis now possible using ML have the potential to greatly increase the scale of ecological assessment on coral reefs. With careful sampling design and evaluation protocols in place, artificial intelligence can serve as a powerful tool for processing this data and unlocking new insights. The potential demonstrated here also has implications for other marine and terrestrial habitats where these techniques could be applied. While there remains much to discover in this field, soundscape ecology has the potential to support our understanding, protection and restoration of coral reefs and other habitats around the world.

## Supporting information

S1 Fig Map of study locations and sites. (A) The location where each dataset was collected. (B) The location of Bontosua and Badi islands, where study sites in the Indonesian dataset were located. (C) The location of the study site on Badi Island. (D) The location of the sites around Bontosua Island. Healthy and degraded sites around Bontosua and Badi Islands are labelled in green and orange respectively. (E) The location of study sites around Lizard Island, where the Australian dataset was collected. High fish diversity sites are labelled in green, low fish diversity sites are labelled in orange and four sites excluded from ecological category tasks are in pink. (F) The location of Mo’orea, Tahiti and Tikehau, where study sites in the French Polynesian dataset were located. (G) The location of study sites around Mo’orea and Tahiti. (H) The location of the study site on Tikehau. Maps were created using the OpenStreetMap base layer (https://www.openstreetmap.org), licensed under the Open Data Commons Open Database License (https://www.openstreetmap.org/copyright).
(DOCX)

S2 Fig Plots showing (A) fish species richness and (B) total fish assemblage biomass recorded from three transect surveys on each of the 12 sites around Lizard Island, Australia. Two ecological categories were first created using the four highest and four lowest scoring sites for species richness, marked in green and orange respectively. These two categories were also found to have non-overlapping biomass values and therefore the categories were labelled as ‘high fish diversity’ and ‘low fish diversity’ sites. The four sites excluded from ecological category tasks are labelled in pink.
(DOCX)

S3 Fig Uniform manifold approximation (UMAP) plots used to represent the compound index, pretrained CNN and trained CNN embeddings in two-dimensional space. Individual points represent a one-minute recording. Plots were produced for each of the Indonesian (A), Australian (B) and French Polynesian (C) datasets and are labelled with colours corresponding to either site or habitat class.
(DOCX)

S4 Fig Interactive UMAP plot of the Indonesia recordings. See S3 Text for more information.
(HTML)

S5 Fig Interactive UMAP plot of the Australia recordings. See S3 Text for more information.
(HTML)

S6 Fig Interactive UMAP plot of the French Polynesia recordings. See S3 Text for more information.
(HTML)

S7 Fig Boxplots of supervised classifier accuracies for the six different tasks across repeated training instances using each of the three embedding extraction methods (compound index, pretrained CNN and trained CNN). Boxes and their bars represent the 25^th^, 50^th^ and 75^th^ quartiles. The black dotted lines represent the expected accuracy using random classification (1/ number of classes). For each task, letters indicate a significant difference between these groupings according to ANOVA. The directions of significant differences reported by the Tukey HSD are indicated, with ‘A’ indicating the group with the highest accuracy, ‘B’ indicating the second highest accuracy, and where present, ‘C’ indicating the third group with the lowest accuracy. N = 100 for the number of repeats performed for all tasks, except for the ‘high or low fish diversity and ‘shallow or mesophotic’ tasks, where N = 32 (S2 Text).
(DOCX)

S8 Fig Confusion matrices pooled across all repeats of trained classifiers for each method and task using the Compound index, Pretrained CNN and Trained CNN on the Indonesian (A), Australian (B) and French Polynesian (C) datasets. True classes are displayed along the x-axis with predicted classes across the y-axis.
(DOCX)

S9 Fig Boxplots of individual acoustic index values for sites from the (A) Indonesian, (B) Australian and (C) French Polynesian datasets. Green boxes indicate high coral cover, high fish diversity and shallow reef classes for the Indonesian, Australian and French Polynesian dataset respectively, with orange indicating the opposing class, and, pink indicating the four sites excluded from habitat category task for the Australian dataset. The index with the highest significant difference between habitat classes reported for each respective dataset was selected for plotting. These were the full band acoustic complexity Index (ACI), normalised difference soundscape index (NDSI), and low band acoustic complexity index (ACI) respectively. Boxes and their bars represent the 25th, 50th and 75th quartile. The overlap of index values across sites prevents the classification of individual sites using this approach.
(DOCX)

S1 Table Three-way ANOVAs comparing supervised classifier accuracy over six different tasks across repeated training instances for the three machine learning methods (compound index, pretrained CNN and trained CNN). Where significant differences (p < 0.05) were detected, post hoc Tukey HSD tests were performed, otherwise cells are left empty. Under the Tukey HSD heading, entries under the first sub-column indicate this method reported a significantly higher accuracy than the method in the second sub-column beneath. 95% Confidence intervals (CI) represent the range of estimated differences in classifier accuracy between the respective pair of methods.
(DOCX)

S1 Text Recording schedules and train, validation, test divisions. (DOCX)

S2 Text Motorboat noise checks. (DOCX)

S3 Text Interactive UMAP plots. (DOCX)
