# Supplementary material for: Unlocking the soundscape of coral reefs with artificial intelligence: pretrained networks and unsupervised learning win out
Source: PLoS Comput Biol. 2025 Apr 28;21(4):e1013029. doi: 10.1371/journal.pcbi.1013029 (PMC12064026; doi:10.1371/journal.pcbi.1013029)
Supplement: S1 Fig — (A) The location where each dataset was collected. (B) The location of Bontosua and Badi islands, where study sites in the Indonesian dataset were located. (C) The location of the study site on Badi Island. (D) The location of the sites around Bontosua Island. Healthy and degraded sites around Bontosua and Badi Islands are labelled in green and orange respectively. (E) The location of study sites around Lizard Island, where the Australian dataset was collected. High fish diversity sites are labelled in green, low fish diversity sites are labelled in orange and four sites excluded from ecological category tasks are in pink. (F) The location of Mo’orea, Tahiti and Tikehau, where study sites in the French Polynesian dataset were located. (G) The location of study sites around Mo’orea and Tahiti. (H) The location of the study site on Tikehau. Maps were created using the OpenStreetMap base layer (https://www.openstreetmap.org), licensed under the Open Data Commons Open Database License (https://www.openstreetmap.org/copyright). (DOCX) [file pcbi.1013029.s001.docx]

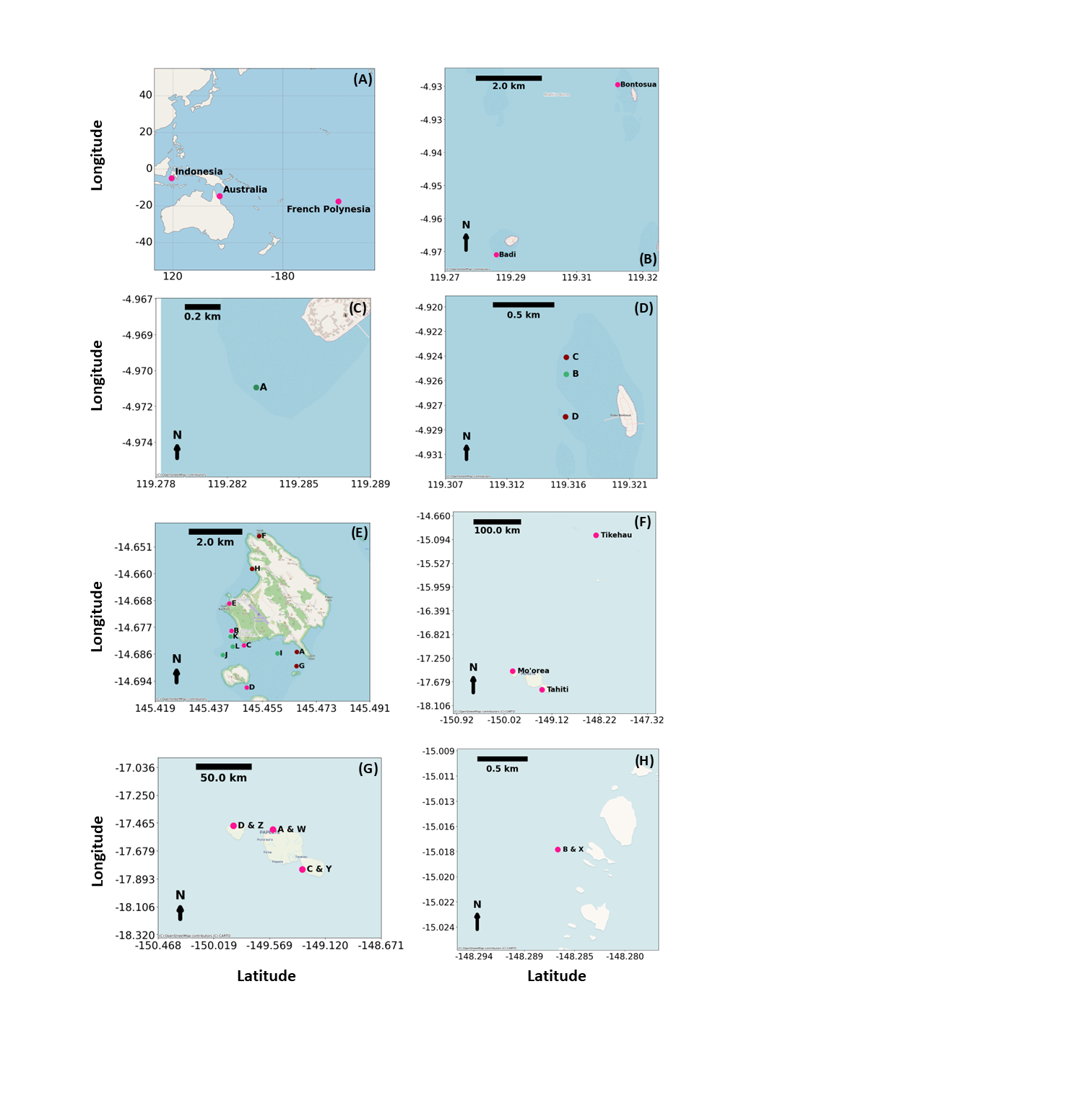


**S1 Fig.** Map of study locations and sites. **(A)** The location where each dataset was collected. **(B)** The location of Bontosua and Badi islands, where study sites in the Indonesian dataset were located. **(C)** The location of the study site on Badi Island. **(D)** The location of the sites around Bontosua Island. Healthy and degraded sites around Bontosua and Badi Islands are labelled in green and orange respectively. **(E)** The location of study sites around Lizard Island, where the Australian dataset was collected. High fish diversity sites are labelled in green, low fish diversity sites are labelled in orange and four sites excluded from ecological category tasks are in pink. **(F)** The location of Mo’orea, Tahiti and Tikehau, where study sites in the French Polynesian dataset were located. **(G)** The location of study sites around Mo’orea and Tahiti. **(H)** The location of the study site on Tikehau. Maps were created using the OpenStreetMap base layer ([https://www.openstreetmap.org](https://eur01.safelinks.protection.outlook.com/?url=https%3A%2F%2Fwww.openstreetmap.org%2F&data=05%7C02%7Cben.williams.20%40ucl.ac.uk%7Cf4330eeffaa54182a93308dd5bec7375%7C1faf88fea9984c5b93c9210a11d9a5c2%7C0%7C0%7C638767793551640222%7CUnknown%7CTWFpbGZsb3d8eyJFbXB0eU1hcGkiOnRydWUsIlYiOiIwLjAuMDAwMCIsIlAiOiJXaW4zMiIsIkFOIjoiTWFpbCIsIldUIjoyfQ%3D%3D%7C0%7C%7C%7C&sdata=PIya3fLpc51lZTXleQxWve1CKqzjkPUOWTE6cfhfnKU%3D&reserved=0)), licensed under the Open Data Commons Open Database License ([https://www.openstreetmap.org/copyright](https://eur01.safelinks.protection.outlook.com/?url=https%3A%2F%2Fwww.openstreetmap.org%2Fcopyright&data=05%7C02%7Cben.williams.20%40ucl.ac.uk%7Cf4330eeffaa54182a93308dd5bec7375%7C1faf88fea9984c5b93c9210a11d9a5c2%7C0%7C0%7C638767793551673931%7CUnknown%7CTWFpbGZsb3d8eyJFbXB0eU1hcGkiOnRydWUsIlYiOiIwLjAuMDAwMCIsIlAiOiJXaW4zMiIsIkFOIjoiTWFpbCIsIldUIjoyfQ%3D%3D%7C0%7C%7C%7C&sdata=bxs9AUlWJ5Ze2uaOM99aKIxW2FlAbZCD1uaPexvEAE4%3D&reserved=0)).
