# Supplementary material for: Unlocking the soundscape of coral reefs with artificial intelligence: pretrained networks and unsupervised learning win out
Source: PLoS Comput Biol. 2025 Apr 28;21(4):e1013029. doi: 10.1371/journal.pcbi.1013029 (PMC12064026; doi:10.1371/journal.pcbi.1013029)
Supplement: S2 Fig — Two ecological categories were first created using the four highest and four lowest scoring sites for species richness, marked in green and orange respectively. These two categories were also found to have non-overlapping biomass values and therefore the categories were labelled as ‘high fish diversity’ and ‘low fish diversity’ sites. The four sites excluded from ecological category tasks are labelled in pink. (DOCX) [file pcbi.1013029.s002.docx]

**
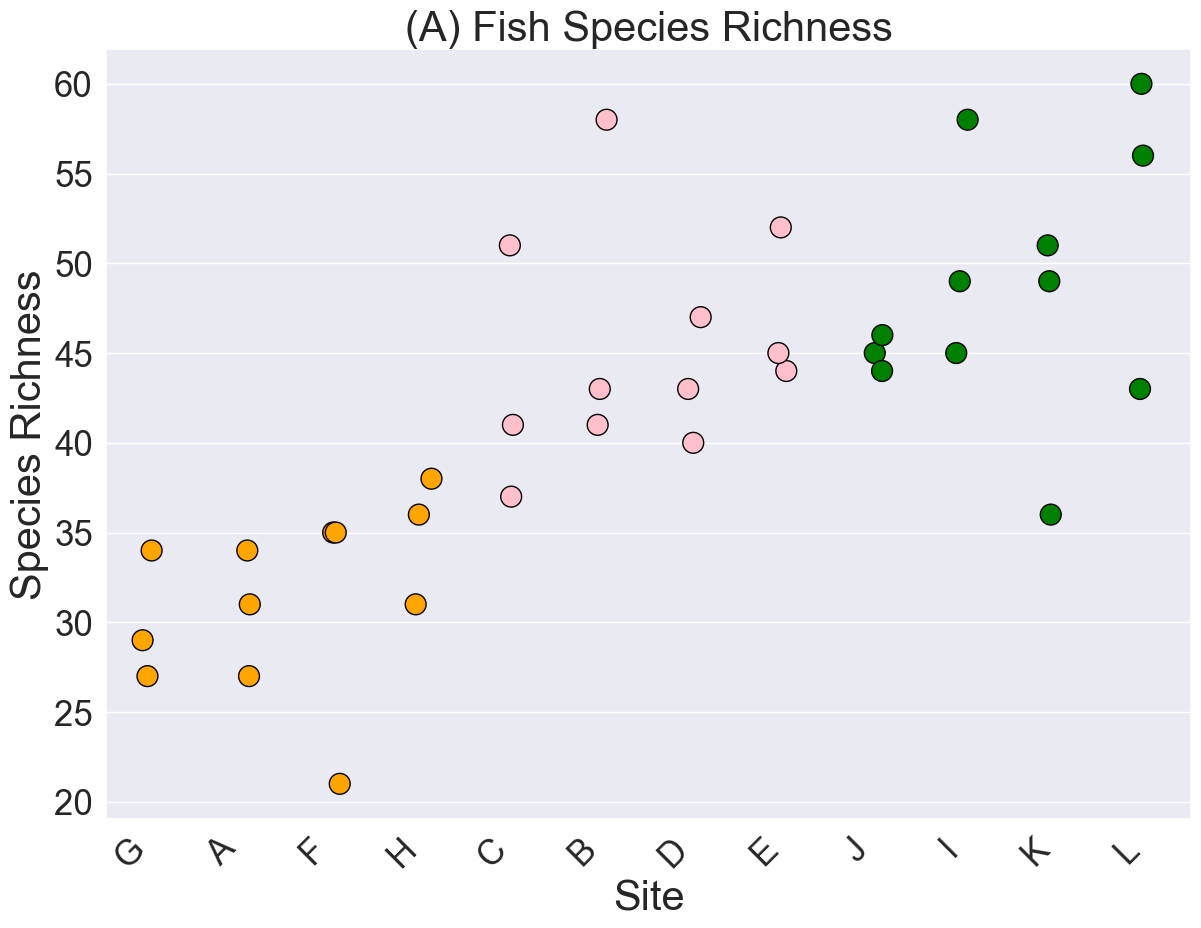

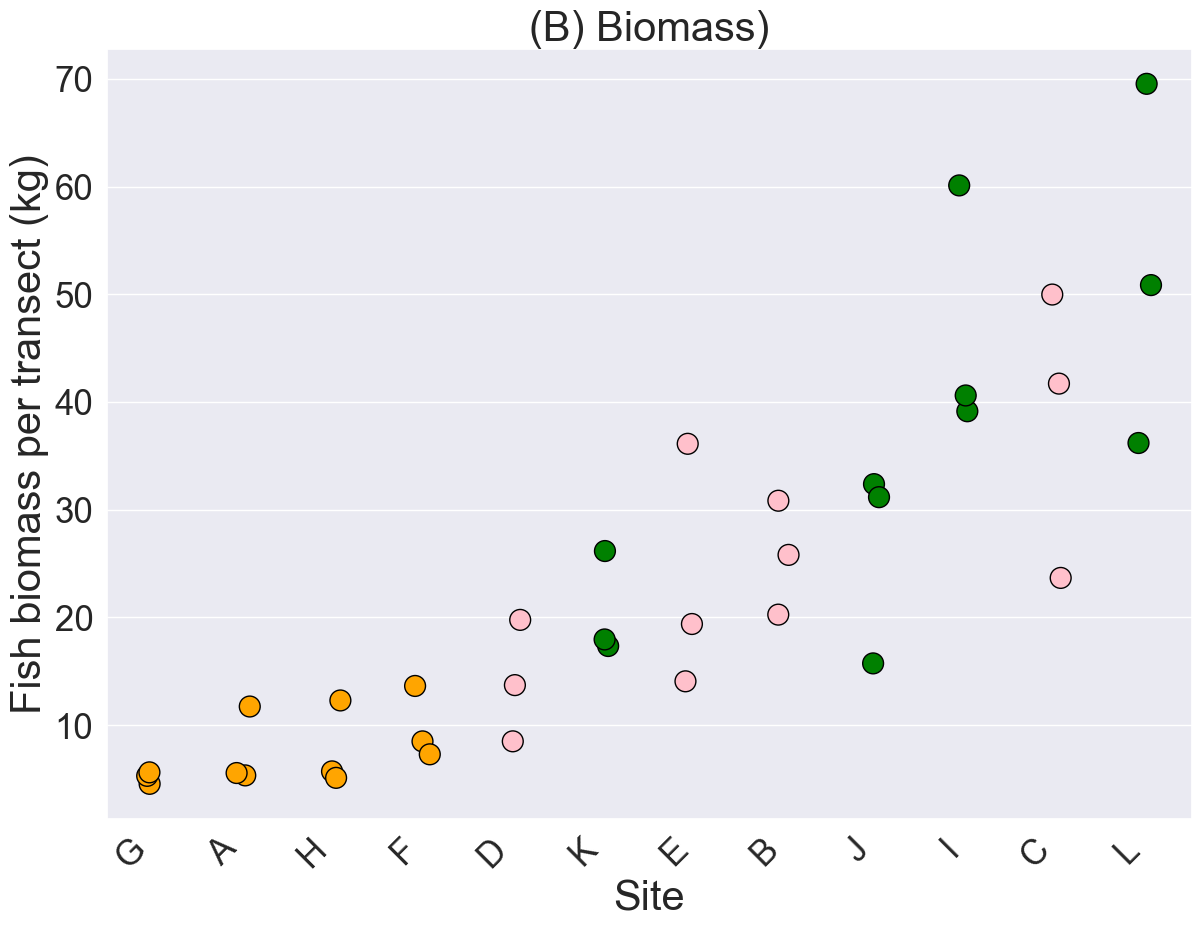
**

**S2 Fig.** Plots showing (A) fish species richness and (B) total fish assemblage biomass recorded from three transect surveys on each of the 12 sites around Lizard Island, Australia. Two ecological categories were first created using the four highest and four lowest scoring sites for species richness, marked in green and orange respectively. These two categories were also found to have non-overlapping biomass values and therefore the categories were labelled as ‘high fish diversity’ and ‘low fish diversity’ sites. The four sites excluded from ecological category tasks are labelled in pink.
