# Supplementary material for: Unlocking the soundscape of coral reefs with artificial intelligence: pretrained networks and unsupervised learning win out
Source: PLoS Comput Biol. 2025 Apr 28;21(4):e1013029. doi: 10.1371/journal.pcbi.1013029 (PMC12064026; doi:10.1371/journal.pcbi.1013029)
Supplement: S3 Fig — Individual points represent a one-minute recording. Plots were produced for each of the Indonesian (A), Australian (B) and French Polynesian (C) datasets and are labelled with colours corresponding to either site or habitat class. (DOCX) [file pcbi.1013029.s003.docx]

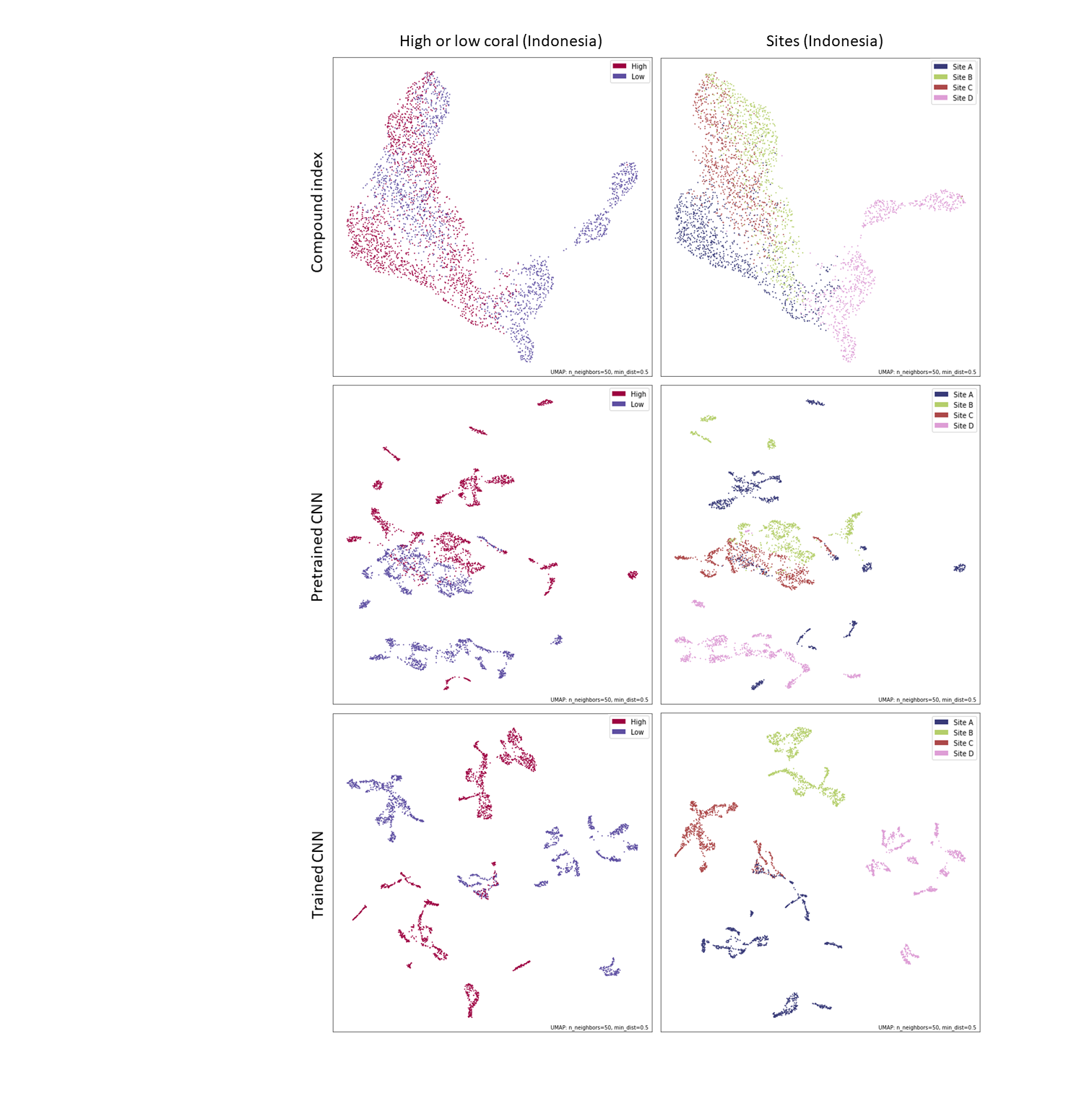


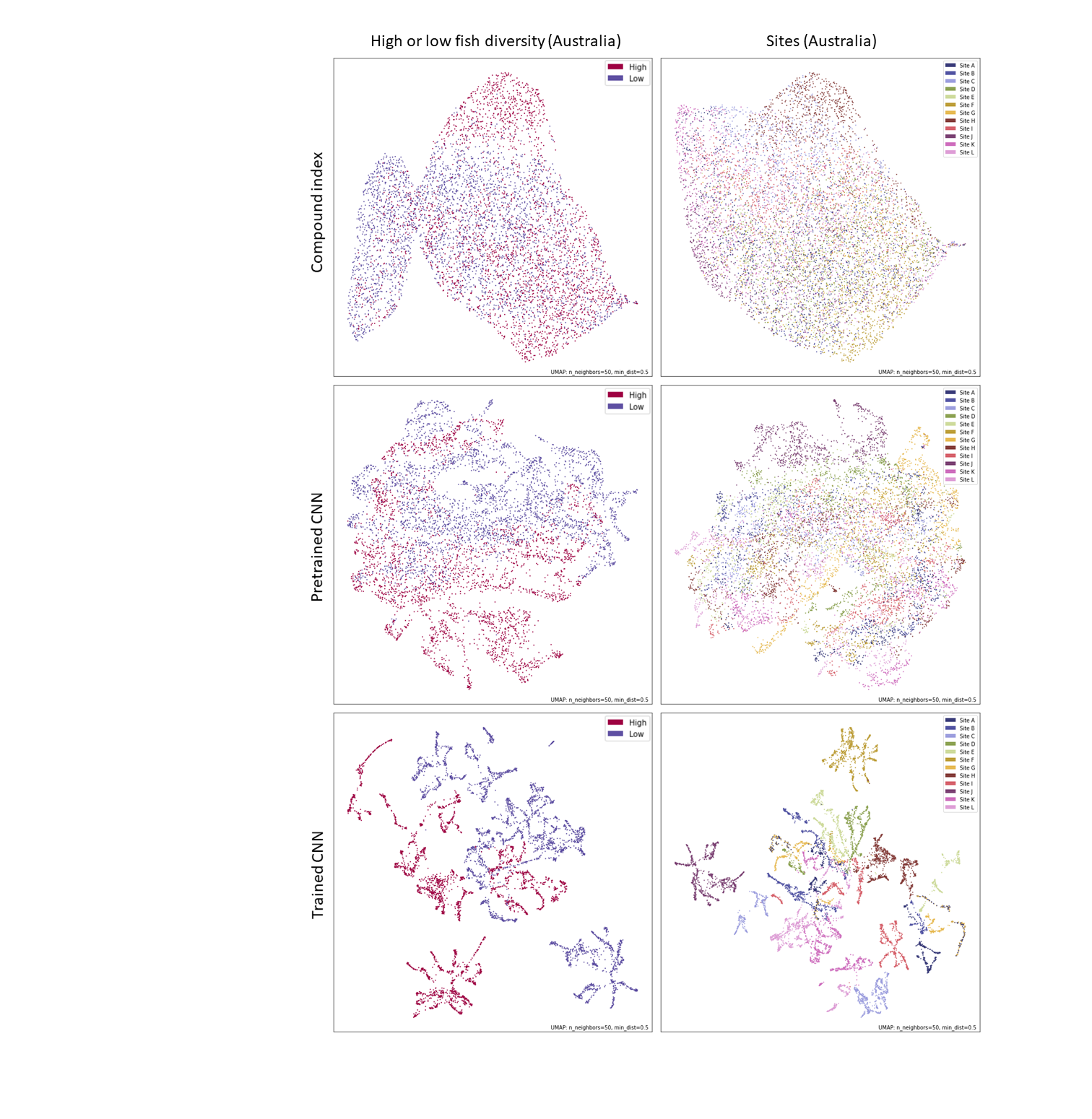


**B**

**
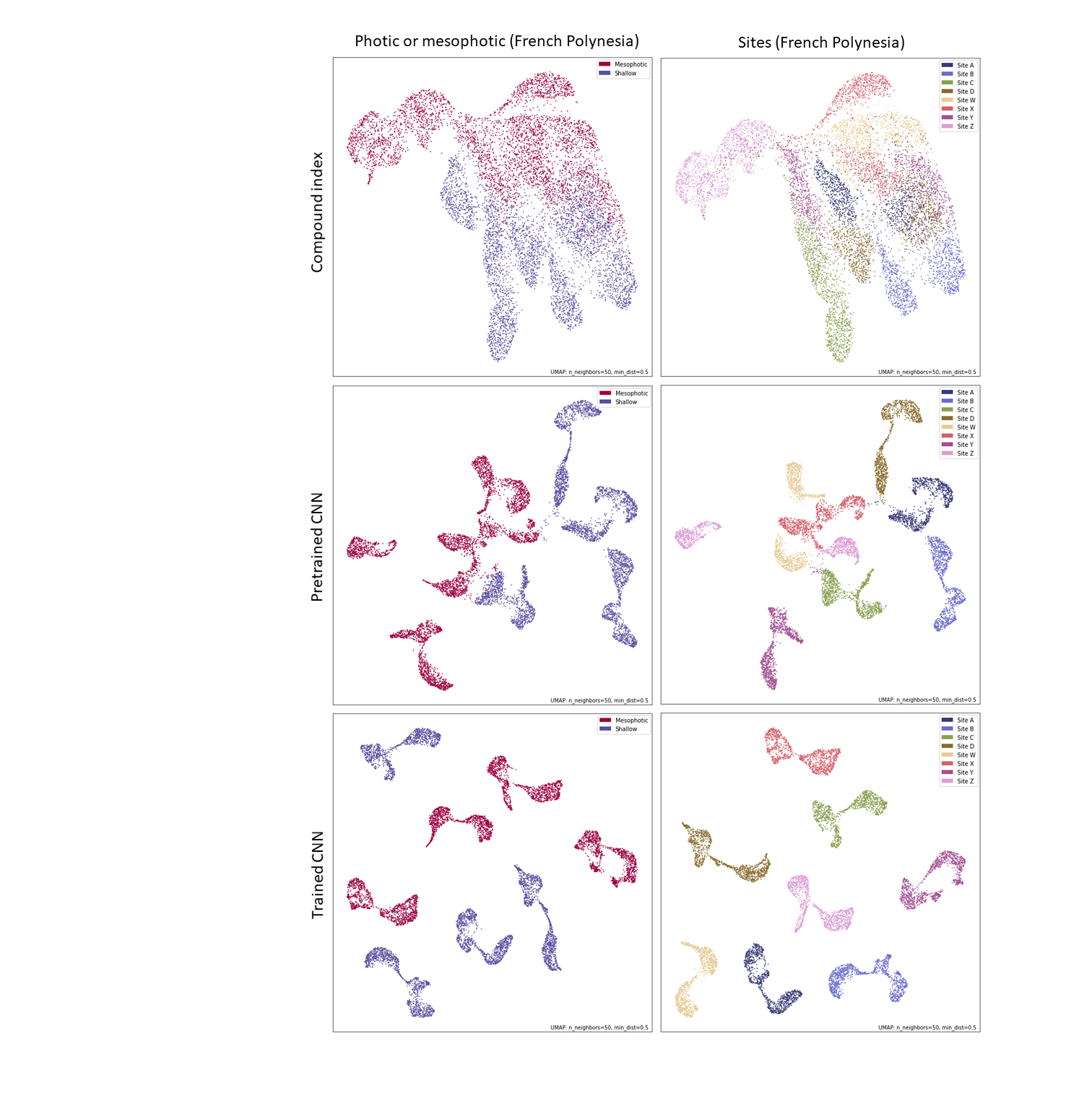
**

**C**

**S3 Fig.** Uniform manifold approximation (UMAP) plots used to represent the compound index, pretrained CNN and trained CNN embeddings in two-dimensional space. Individual points represent a one-minute recording. Plots were produced for each of the Indonesian (A), Australian (B) and French Polynesian (C) datasets and are labelled with colours corresponding to either site or habitat class.
