# Supplementary material for: Unlocking the soundscape of coral reefs with artificial intelligence: pretrained networks and unsupervised learning win out
Source: PLoS Comput Biol. 2025 Apr 28;21(4):e1013029. doi: 10.1371/journal.pcbi.1013029 (PMC12064026; doi:10.1371/journal.pcbi.1013029)
Supplement: S7 Fig — Boxes and their bars represent the 25th, 50th and 75th quartiles. The black dotted lines represent the expected accuracy using random classification (1/ number of classes). For each task, letters indicate a significant difference between these groupings according to ANOVA. The directions of significant differences reported by the Tukey HSD are indicated, with ‘A’ indicating the group with the highest accuracy, ‘B’ indicating the second highest accuracy, and where present, ‘C’ indicating the third group with the lowest accuracy. N = 100 for the number of repeats performed for all tasks, except for the ‘high or low fish diversity and ‘shallow or mesophotic’ tasks, where N = 32 (S2 Text). (DOCX) [file pcbi.1013029.s007.docx]

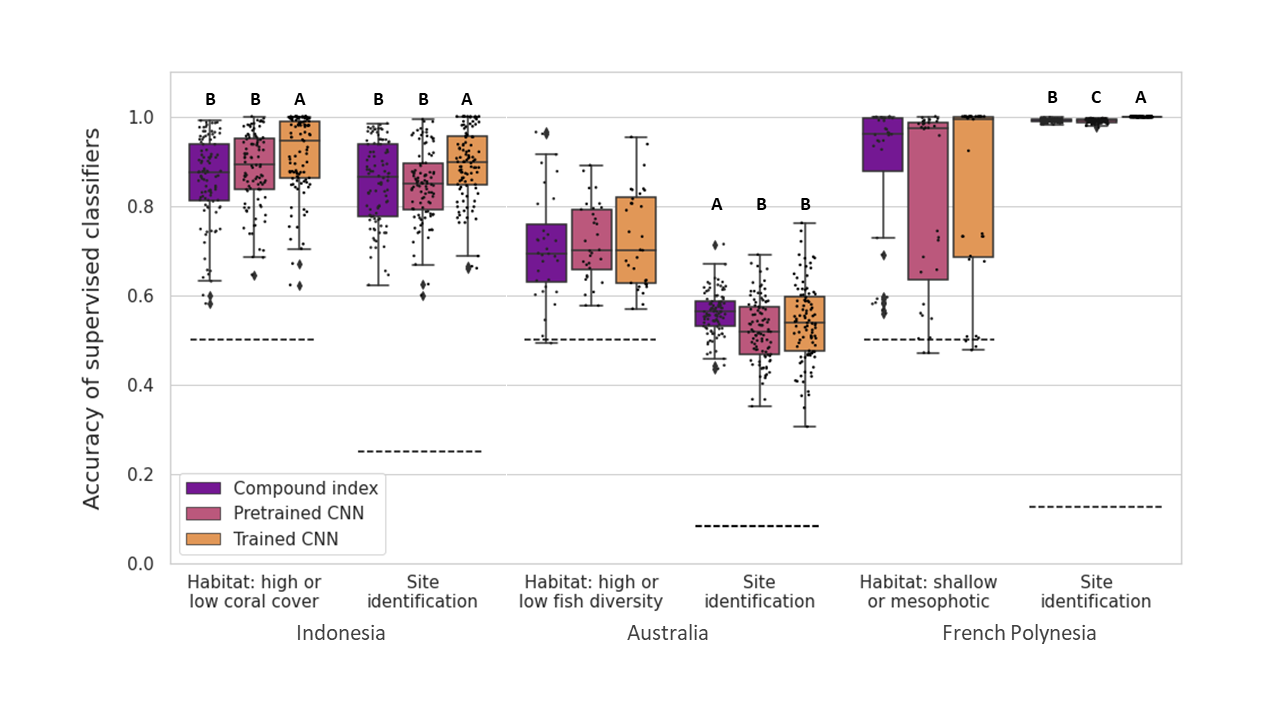


**S7 Fig**. Boxplots of supervised classifier accuracies for the six different tasks across repeated training instances using each of the three embedding extraction methods (compound index, pretrained CNN and trained CNN). Boxes and their bars represent the 25^th^, 50^th^ and 75^th^ quartiles. The black dotted lines represent the expected accuracy using random classification (1 / number of classes). For each task, letters indicate a significant difference between these groupings according to ANOVA. The directions of significant differences reported by the Tukey HSD are indicated, with ‘A’ indicating the group with the highest accuracy, ‘B’ indicating the second highest accuracy, and where present, ‘C’ indicating the third group with the lowest accuracy. N = 100 for the number of repeats performed for all tasks, except for the ‘high or low fish diversity and ‘shallow or mesophotic’ tasks, where N = 32 (S2 Text).
