# Supplementary material for: Unlocking the soundscape of coral reefs with artificial intelligence: pretrained networks and unsupervised learning win out
Source: PLoS Comput Biol. 2025 Apr 28;21(4):e1013029. doi: 10.1371/journal.pcbi.1013029 (PMC12064026; doi:10.1371/journal.pcbi.1013029)
Supplement: S8 Fig — True classes are displayed along the x-axis with predicted classes across the y-axis. (DOCX) [file pcbi.1013029.s008.docx]

**A**

**High or low coral cover (Indonesia)**

**Site identification (Indonesia)**


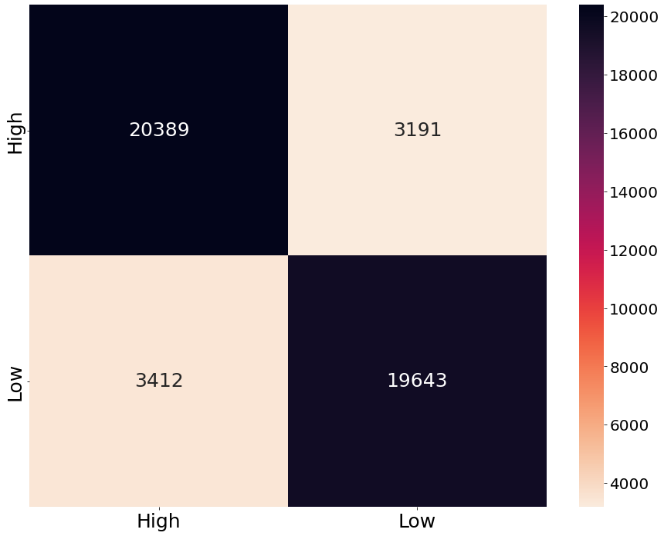

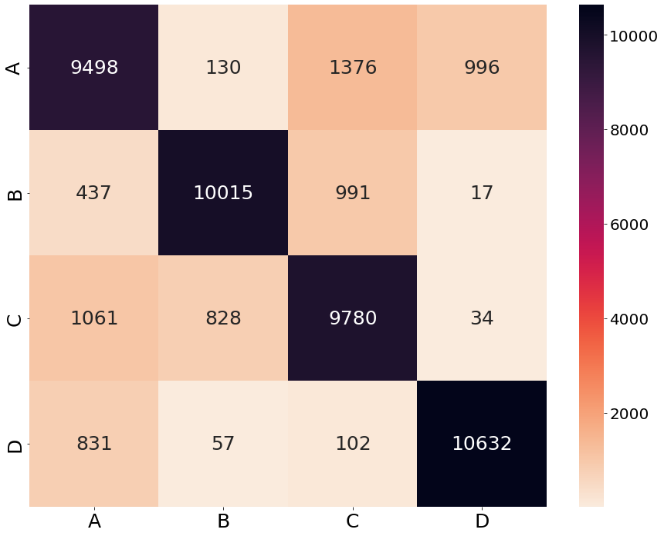


**Compound index**


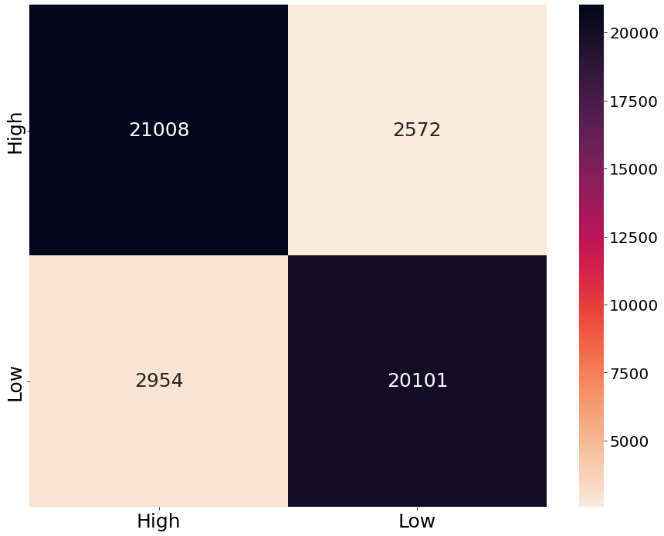

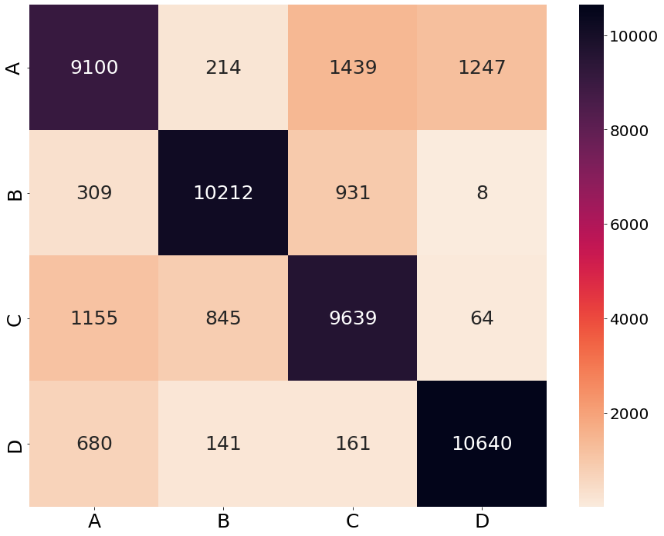


**Pretrained CNN**


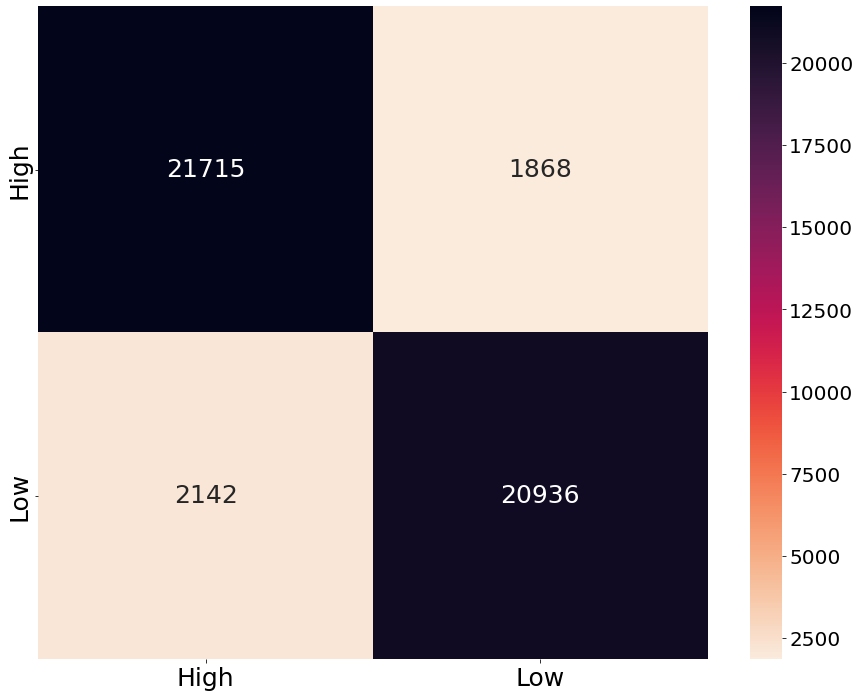

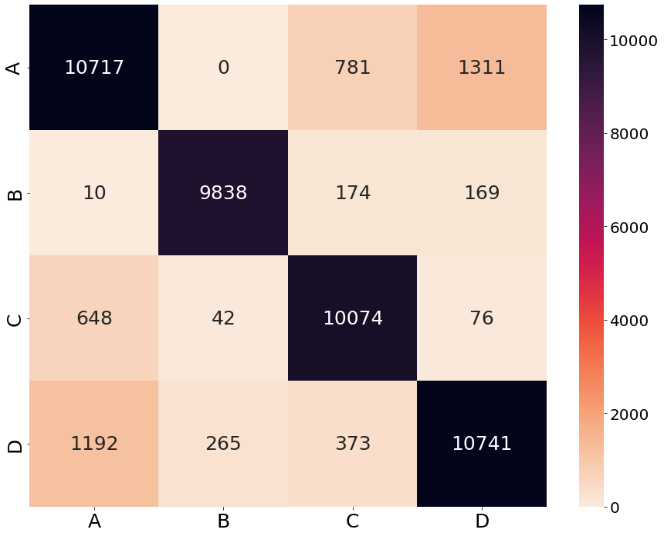


**Trained CNN**

**B**

**Site identification (Australia)**

**High or low fish diversity (Australia)**


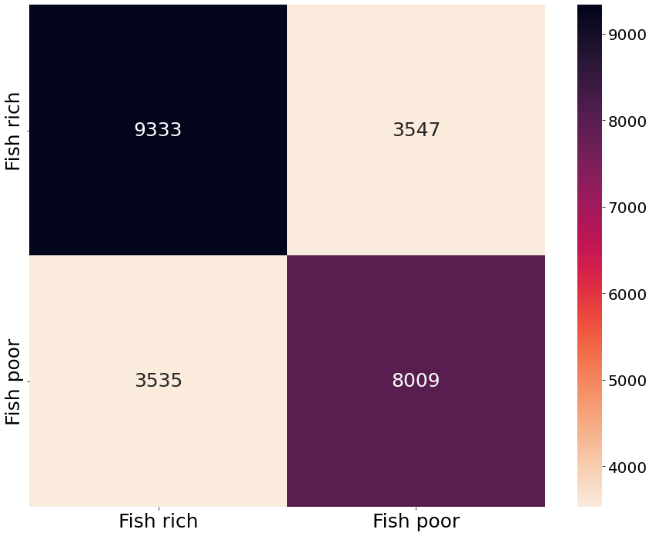

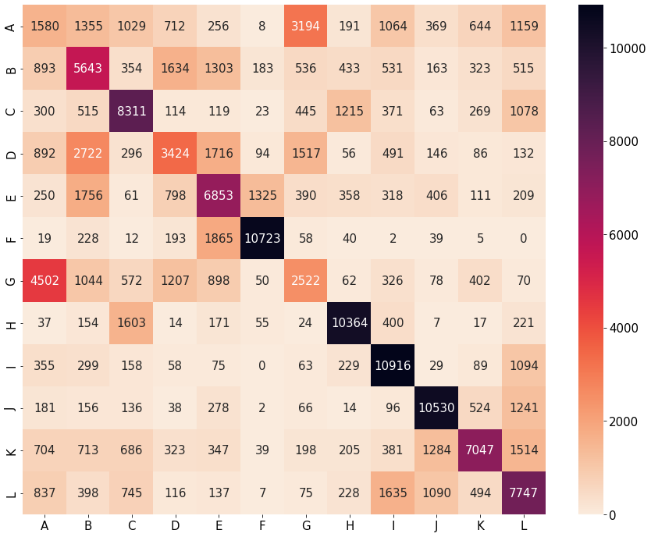


**Compound index**


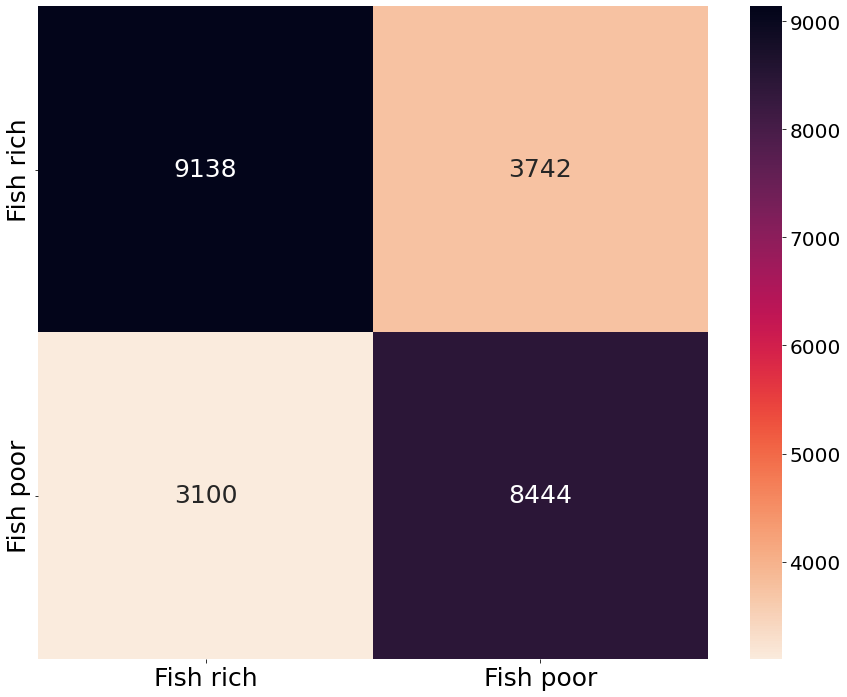

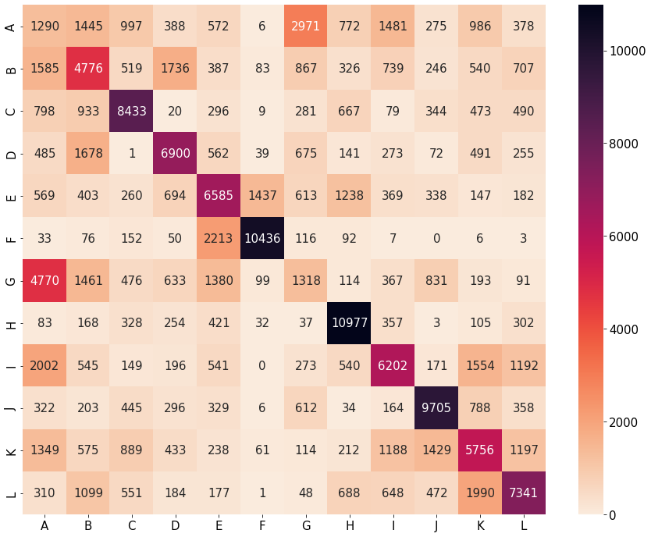


**Pretrained CNN**


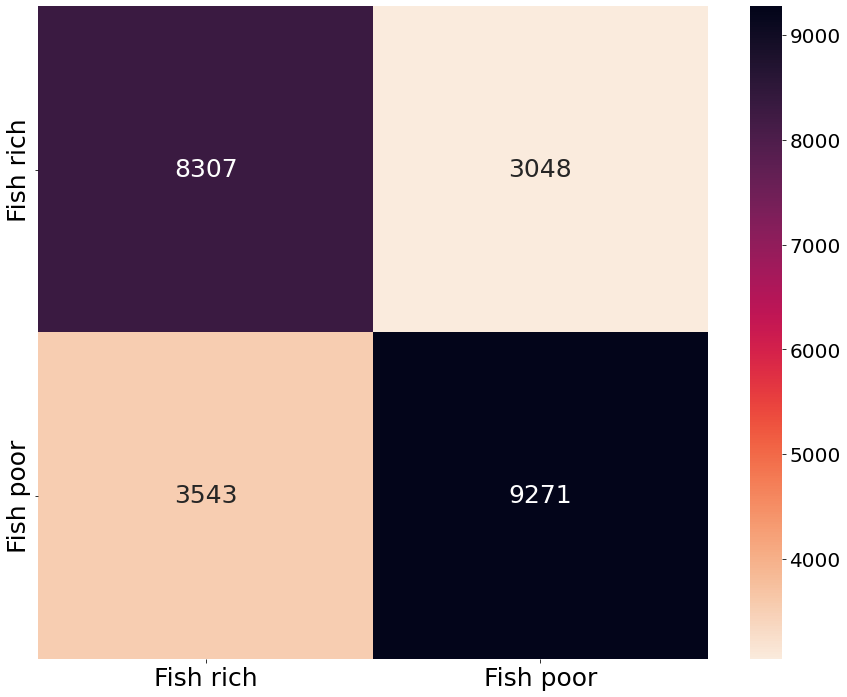

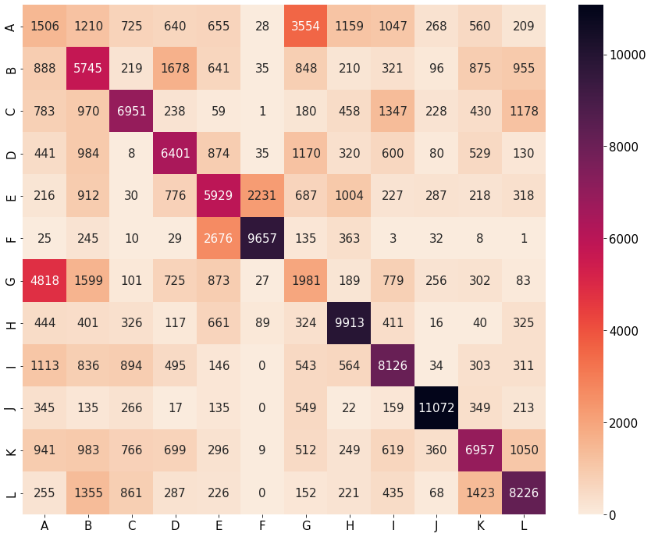


**Trained CNN**

**C**

**Site identification (French Polynesia)**

**Shallow or mesophotic (French Polynesia)**


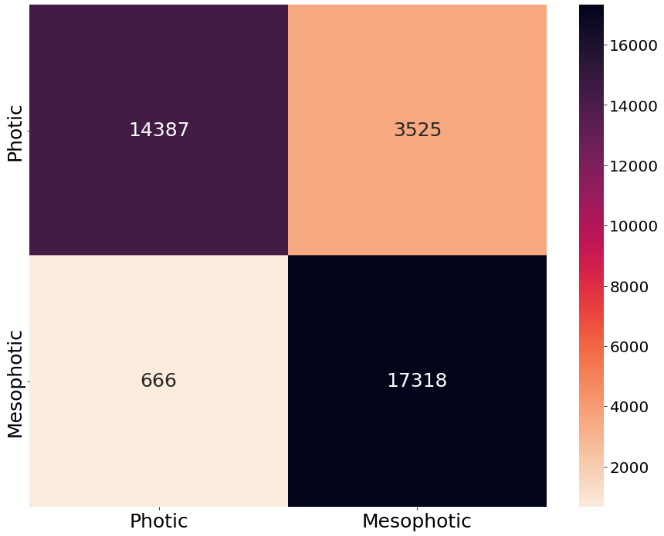

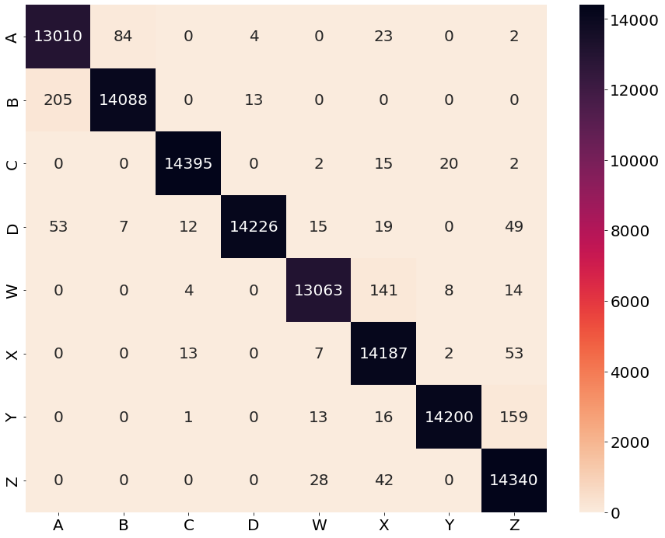


**Compound index**


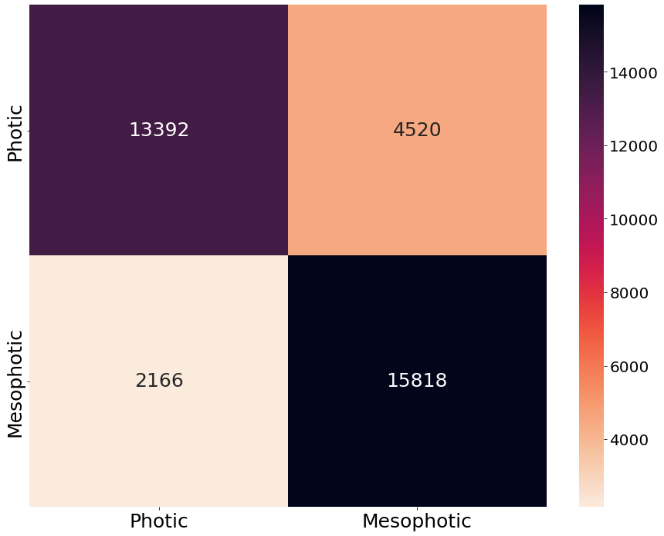

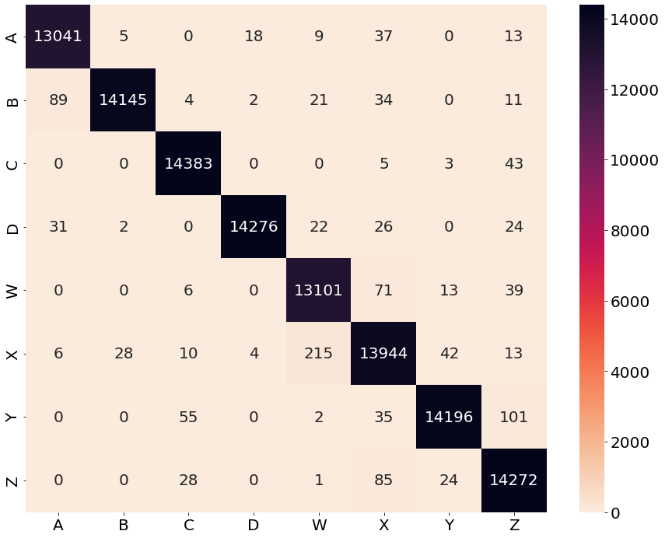


**Pretrained CNN**


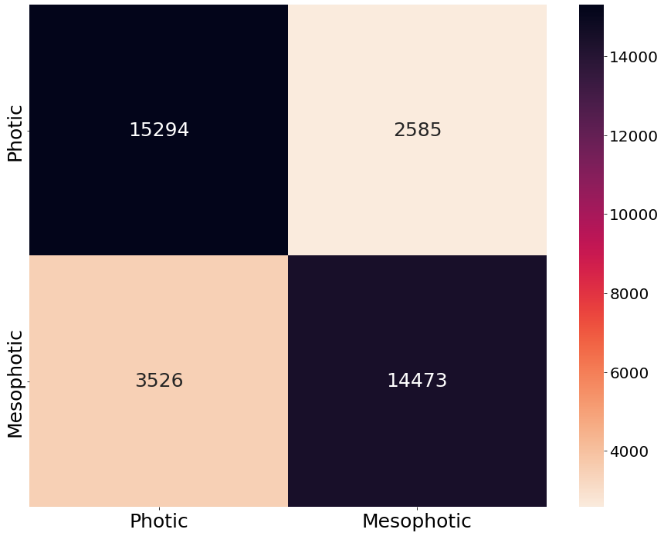

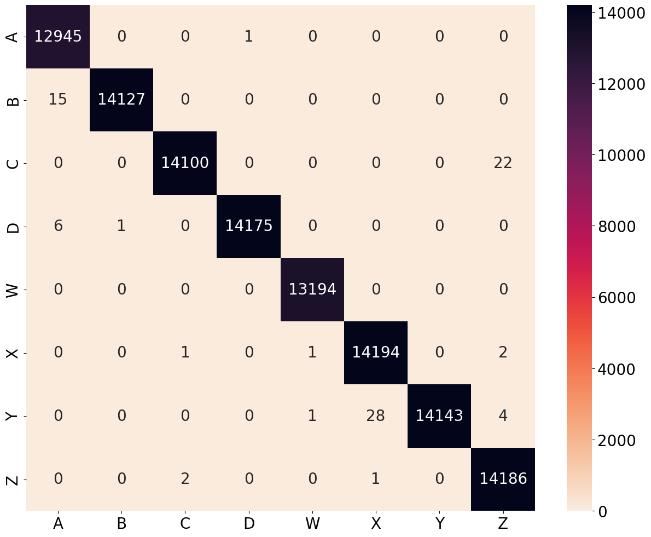


**Trained CNN**

**S8 Fig.** Confusion matrices pooled across all repeats of trained classifiers for each method and task using the Compound index, Pretrained CNN and Trained CNN on the Indonesian (A), Australian (B) and French Polynesian (C) datasets. True classes are displayed along the x-axis with predicted classes across the y-axis.
