# Supplementary material for: Unlocking the soundscape of coral reefs with artificial intelligence: pretrained networks and unsupervised learning win out
Source: PLoS Comput Biol. 2025 Apr 28;21(4):e1013029. doi: 10.1371/journal.pcbi.1013029 (PMC12064026; doi:10.1371/journal.pcbi.1013029)
Supplement: S9 Fig — Green boxes indicate high coral cover, high fish diversity and shallow reef classes for the Indonesian, Australian and French Polynesian dataset respectively, with orange indicating the opposing class, and, pink indicating the four sites excluded from habitat category task for the Australian dataset. The index with the highest significant difference between habitat classes reported for each respective dataset was selected for plotting. These were the full band acoustic complexity Index (ACI), normalised difference soundscape index (NDSI), and low band acoustic complexity index (ACI) respectively. Boxes and their bars represent the 25th, 50th and 75th quartile. The overlap of index values across sites prevents the classification of individual sites using this approach. (DOCX) [file pcbi.1013029.s009.docx]

**
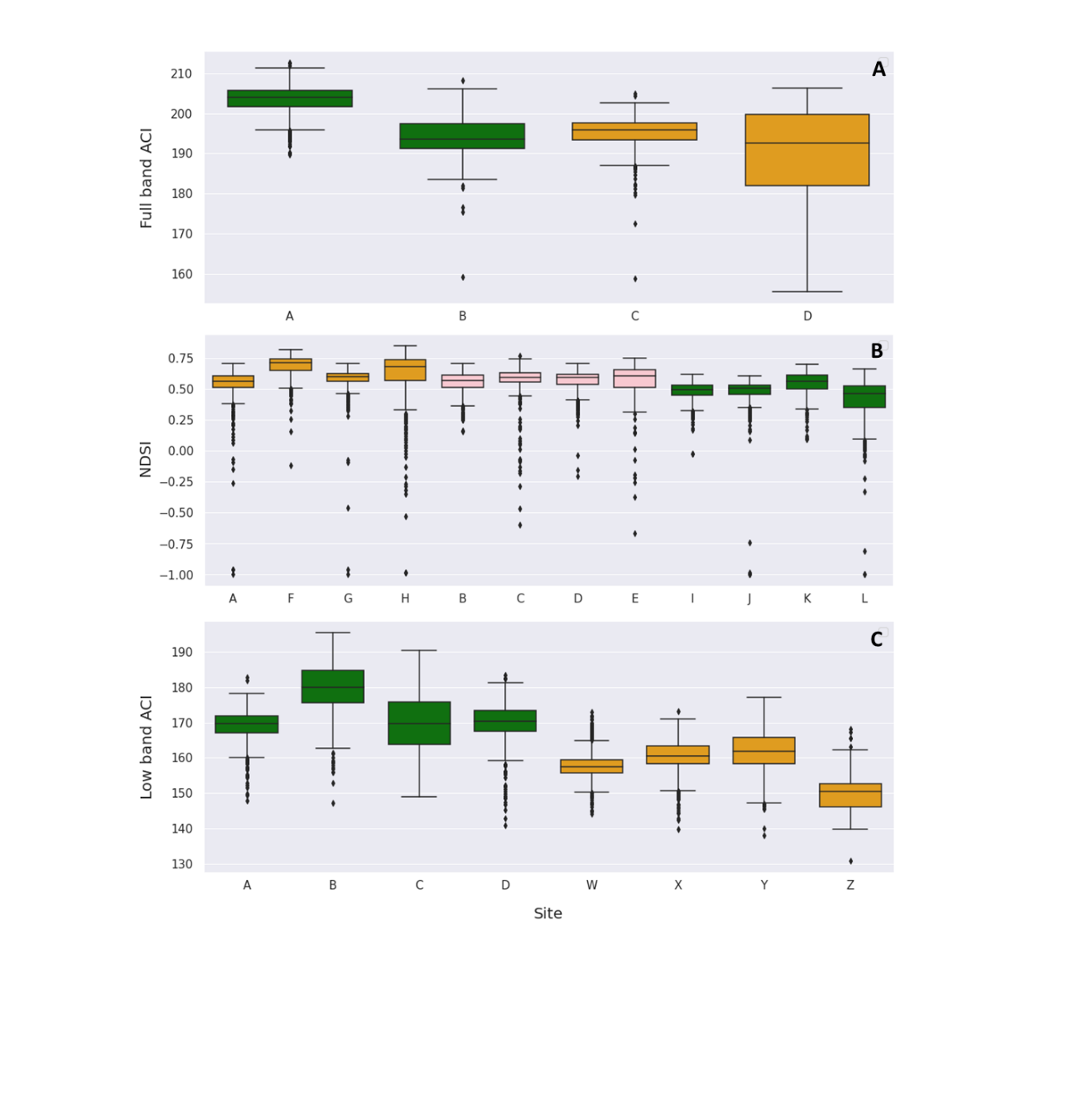
**

**S9 Fig.** Boxplots of individual acoustic index values for sites from the **(A)** Indonesian, **(B)** Australian and **(C)** French Polynesian datasets. Green boxes indicate high coral cover, high fish diversity and shallow reef classes for the Indonesian, Australian and French Polynesian dataset respectively, with orange indicating the opposing class, and, pink indicating the four sites excluded from habitat category task for the Australian dataset. The index with the highest significant difference between habitat classes reported for each respective dataset was selected for plotting. These were the full band acoustic complexity Index (ACI), normalised difference soundscape index (NDSI), and low band acoustic complexity index (ACI) respectively. Boxes and their bars represent the 25th, 50th and 75th quartile. The overlap of index values across sites prevents the classification of individual sites using this approach.
