# Supplementary material for: Unlocking the soundscape of coral reefs with artificial intelligence: pretrained networks and unsupervised learning win out
Source: PLoS Comput Biol. 2025 Apr 28;21(4):e1013029. doi: 10.1371/journal.pcbi.1013029 (PMC12064026; doi:10.1371/journal.pcbi.1013029)
Supplement: S1 Table — Where significant differences (p < 0.05) were detected, post hoc Tukey HSD tests were performed, otherwise cells are left empty. Under the Tukey HSD heading, entries under the first sub-column indicate this method reported a significantly higher accuracy than the method in the second sub-column beneath. 95% Confidence intervals (CI) represent the range of estimated differences in classifier accuracy between the respective pair of methods. (DOCX) [file pcbi.1013029.s010.docx]

**S1 Table**. Three-way ANOVAs comparing supervised classifier accuracy over six different tasks across repeated training instances for the three machine learning methods (compound index, pretrained CNN and trained CNN). Where significant differences (p<0.05) were detected, post hoc Tukey HSD tests were performed, otherwise cells are left empty. Under the Tukey HSD heading, entries under the first sub-column indicate this method reported a significantly higher accuracy than the method in the second sub-column beneath. 95% Confidence intervals (CI) represent the range of estimated differences in classifier accuracy between the respective pair of methods.

|  |  | **ANOVA result** | | **Tukey HSD results** | | | | | |
| --- | --- | --- | --- | --- | --- | --- | --- | --- | --- |
|  |  |  |  | **Compound index** | | **Pretrained CNN** | | **CNN** | |
| **Dataset** | **Task** | **f-value** | **p-value** | **Pretrained  CNN** | **CNN** | **Compound  index** | **CNN** | **Compound  index** | **Pretrained  CNN** |
| Indonesia | Habitat: high or low coral cover | 9.825 | <0.001 |  |  |  |  | p = 0.001, CI = 0.026 to 0.085 | p = 0.027, CI = 0.003 to 0.062 |
| Indonesia | Site  identification | 8.939 | <0.001 |  |  |  |  | p = 0.003, CI = 0.012 to 0.067 | p = 0.001, CI = 0.019 to 0.074 |
| Australia | Habitat: fish rich or poor | 0.180 | 0.836 |  |  |  |  |  |  |
| Australia | Site  identification | 7.495 | 0.001 | p = 0.001, CI = 0.015 to 0.063 |  |  |  |  |  |
| French Polynesia | Habitat: shallow or mesophotic | 1.143 | 0.323 |  |  |  |  |  |  |
| French Polynesia | Site  identification | 268.343 | <0.001 | p = 0.011, CI = 0.0003 to 0.002 |  |  |  | p = 0.001, CI = 0.007 to 0.0095 | p = 0.011, CI = 0.009 to 0.01 |
