# Supplementary material for: Unlocking the soundscape of coral reefs with artificial intelligence: pretrained networks and unsupervised learning win out
Source: PLoS Comput Biol. 2025 Apr 28;21(4):e1013029. doi: 10.1371/journal.pcbi.1013029 (PMC12064026; doi:10.1371/journal.pcbi.1013029)
Supplement: S1 Text — (DOCX) [file pcbi.1013029.s011.docx]

**S1 Text: Recording schedules and train, validation, test divisions**

Recordings were taken in multiple discrete blocks. Across each dataset, hydrophone recorders were frequently rotated between sites for a new recording block to mitigate against the introduction of instrument bias. For habitat classification tasks, entire sites were excluded from the training data for the Australian and French Polynesian dataset so that unseen sites were evaluated upon. The Indonesian dataset only had two sites in each category, so entire recording blocks were excluded for this. For site classification tasks, entire recording blocks were excluded from the training data and used to evaluate upon. This was to mitigate against temporal autocorrelation, meaning a classifier would not be able to train upon recording periods immediately adjacent to those in the evaluation set. For any given dataset, the same train/validation/test sets combinations were conserved across compound index, P-CNN and T-CNN training. Further details on how this was implemented for each dataset are below.

**Indonesia**

The Indonesian dataset was collected as part of the monitoring programme of the Mars Coral Reef Restoration Project ([www.buildingcoral.com](http://www.buildingcoral.com)). This monitoring programme included recordings collected from four study sites across two reefs (S1B Fig); two high coral cover reefs and two low coral cover reefs (S1B, S1C and S1D Fig). All sites were at a depth between 2–3.3m at low tide. One-hour blocks of soundscape recordings were taken at each site for five days either side of the full moon (26 August 2018) and three days either side of the following new moon (10 September 2018) during daylight (09:00–15:00), twilight (05:30–06:30 and 17:30–18:30) and night-time (23:30–00:30) periods. Handling recorder bias was paramount to the sampling regime design. Data collection was therefore performed in one-hour recording blocks, instead of using longer continuous unbroken recording periods, which allowed recorders to be rotated between sites for each new recording block. These recordings were made in a counterbalanced blocking design, such that there was a similar number of recordings taken from each site (Fig. S1). This design also factored in an approximately even spread of time points and lunar phases taken across sites (see Lamont et al., 2021 (1) for more detail). Recordings were split into one-minute segments, providing a total of 3,335 one-minute recordings.

For the Indonesian habitat and site level classification training and test sets, 57 one-hour recording blocks were available, split approximately evenly across the four sites (Fig. A) (see Lamont et al., 2021 (1)for further details).

1. For the habitat classification task, four blocks were randomly selected from each of the two classes as validation data, and another four as test data, remaining blocks were used as training. The remaining 41 blocks were used for the training set. A new train/val/test combination was selected for each of the 100 training run repeats performed (see Fig. A for one example combination).
2. For the site identification task, validation and test data selection was identical to the habitat task except two blocks were randomly selected from each of the four sites as validation data, and another four as test data (see Fig. A for one example combination).


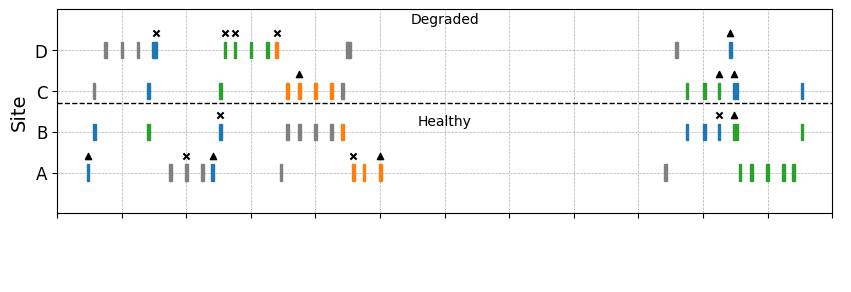

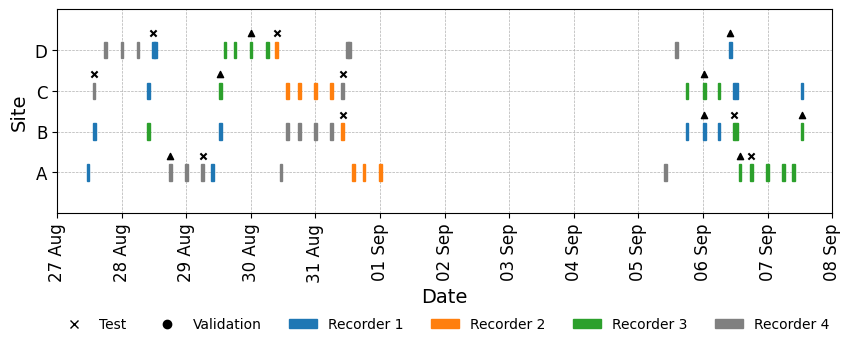


**Fig A.** Plots depicting the recording schedule and instrument rotation to mitigate recorder bias followed for the Indonesian dataset. Gridlines represent midnight. Recordings were taken after the full moon (Aug 26^th^) and before the new moon (Sep 10^th^). One example of the validation and test sets selected for one of the 100 training run repeats performed for the habitat identification task (top) and site identification task (bottom) are indicated by the shapes above blocks.

**Australia**

The Australian dataset consisted of recordings from 12 randomly selected sites (A–L) located around Lizard Island on the Great Barrier Reef; sites were separated by at least 500 m and constituted >250 x 5 m of contiguous reef (S1E Fig). All sites were at a depth between 2–3m at low tide. Six recording blocks of 19–24 hr duration were taken at each site between 23 October to 14 December 2018; blocks from each site were spaced 9 days (SE: ±3.49) apart on average. Recordings were not made during the Nov 11-20^th^ period due to elevated sea states which could introduce geophonic noise. Handling recorder bias was paramount to the sampling regime design. Data collection was therefore performed in these approximately day-long recording blocks, instead of using longer continuous unbroken recording periods, which allowed recorders to be rotated between sites for each new recording block (Fig B).

The final dataset was subsampled to take one-minute clips separated by nine minutes of unused recording, providing 8,127 one-minute recordings in total. Fish community biomass and species richness data were collected along three transects at each site during this period following methods from Richardson et al., (2018). Of the 12 sites, the four with the highest and four with the lowest species richness values were identified and split into two classes, totalling 6289 one-minute recordings after excluding the other four sites (Fig B). The sites from these two classes were also found to have non-overlapping fish biomass values and were therefore split into high and low fish diversity classes.

1. For the habitat level classification training and test divisions, 48 19-24 hr recordings blocks were available after the four sites not in use for the habitat task were removed. For each repeat of a model training run, one entire site was taken from each of the two classes and entire recording blocks from these sites were randomly placed into either the validation or testing set, whilst maintaining a balanced class ratio between the two sets (i.e both the validation and test set always had four blocks each). This was then repeated, with the validation and test set flipped to provide another repeat. This was repeated for every combination of the eight sites, maintaining one high and one low fish diversity site in the validation and training set each time, producing 32 replicates in total (see Fig. B for one example combination).
2. For the site level classification all 12 sites were included, providing 72 19-24 hr recording blocks split evenly across these sites (Fig B). For each repeat of a training run, one recording block was randomly selected from each site as the validation data, and another block for the test data. Remaining blocks were used as training data. A new combination was selected for each of the 100 repeats performed for this task (see Fig. B for one example combination).


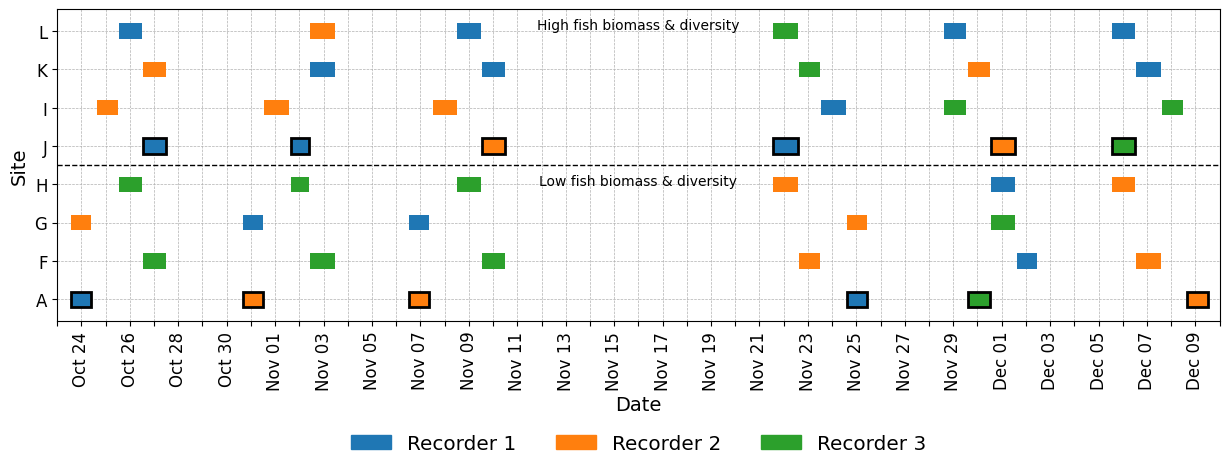

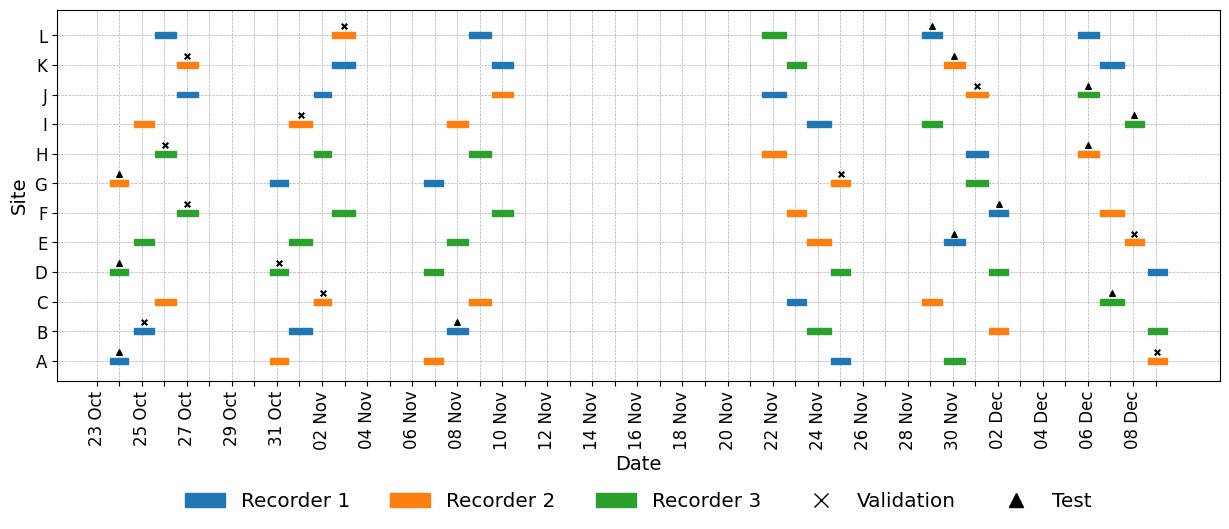


**Fig B.** Plots depicting the recording schedule and instrument rotation to mitigate recorder bias followed for the Australian dataset. Gridlines represent midnight. (Top) One example of the validation and test sets selected for one of the 100 training run repeats performed for the habitat identification task are depicted by the blocks with a black outline. This also shows how data from multiple recorders was selected for each repeat to handle instrument bias. (Bottom) One example of the validation and test sets, selected for one of the 100 training run repeats performed for the site identification task, where shapes above blocks indicate those which were used for the test or validation in this repeat. This also shows how data from multiple recorders was selected for each repeat to handle instrument bias.

**French Polynesia**

The French Polynesian dataset was collected in February and March 2021 at four sites, consisting of reefs around the islands of Mo’orea, Tikehau and Tahiti (S5F, S5G and S5H Figs). At each site, soundscape recordings were taken at a shallow (10–15 m depth) and mesophotic (55–65 m depth) site, totalling four shallow sites (A–D) and four mesophotic sites (W–Z). Recordings were taken simultaneously for each shallow–mesophotic pair for a continuous block of 89–97 hrs from each. Recordings were subsampled to take one-minute clips separated by four minutes of unused recording, providing 8,975 one-minute recordings in total.

1. For the habitat level classification training and test divisions, each repeat of a training run took all recordings from two sites (one from each class). For each repeat, all the recordings from these sites were randomly placed into either the validation or test set, whilst maintaining a balanced class ratio between both sets. The validation and test set were then flipped to add an extra training run repeat. This was performed for every combination of the eight sites, maintaining one shallow and one mesophotic site in the validation and training set each time, producing 32 replicates in total (see Fig C for one example combination).
2. For the site classification task, each repeat of a training run took one contiguous 24 hr recording block from all eight sites for validation or testing data. All recording from the selected 24 hr periods of each site were evenly placed into either the validation or the testing data. A new random combination was taken for each of the 100 repeats performed (see Fig C for one example combination).


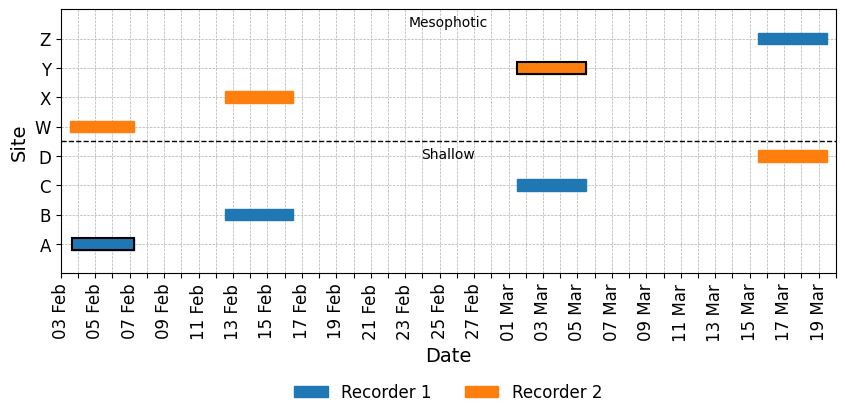

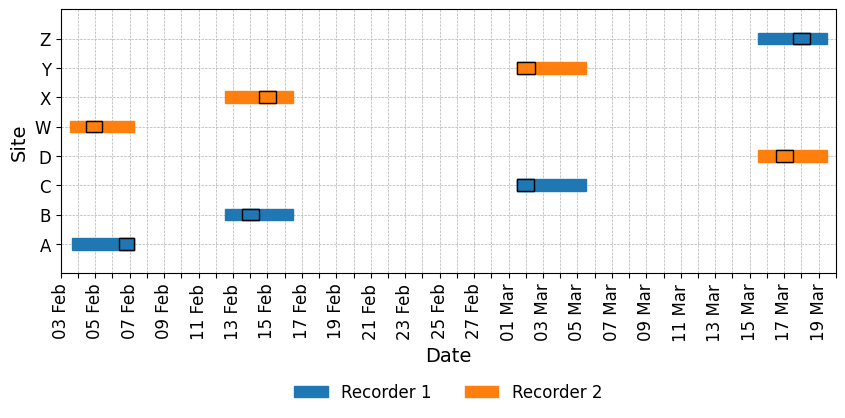


**Fig C.** Plots depicting the recording schedule and instrument rotation followed for the French Polynesian dataset. Gridlines represent midnight. One example of the data randomly set aside for validation and testing of a given training run repeat is shown depicted by the black outlines for the habitat (top) and site task (bottom).

**References**

1. Lamont TA, Williams B, Chapuis L, Prasetya ME, Seraphim MJ, Harding HR, May EB, Janetski N, Jompa J, Smith DJ, Radford AN. The sound of recovery: Coral reef restoration success is detectable in the soundscape. J. Appl. Ecol. 2022;59: 742-56.
