# Supplementary material for: Unlocking the soundscape of coral reefs with artificial intelligence: pretrained networks and unsupervised learning win out
Source: PLoS Comput Biol. 2025 Apr 28;21(4):e1013029. doi: 10.1371/journal.pcbi.1013029 (PMC12064026; doi:10.1371/journal.pcbi.1013029)
Supplement: S2 Text — (DOCX) [file pcbi.1013029.s012.docx]

**S2 Text: Motorboat noise checks**

If extensive boat noise were present in some sites or habitat types but not others, this could enable models to learn features relevant to boat noise and not the biophony. Boat noise is documented to interact with elements of the ecology on reefs^1–3^ which may make its presence in the soundscape an important indicator. Future studies should consider the removal or inclusion of boat noise carefully.

To check for excessive boat noise, two-hundred one-minute recordings were sampled from each dataset, evenly distributed across sites but otherwise at random. The first 30-seconds of each recording were checked for boat noise by BW, totalling 5hrs of data. None of the samples from the Indonesian dataset contained boat noise. Two samples from both the Australian and French Polynesian datasets contained boat noise, from site D and G, and, site W and A respectively. This indicates approximately 1% of the data contained boat noise. If boat noise is not evenly distributed across sites the models would still have to learn features from the biophony for 99% of samples.

**References**

1. Ferrier-Pagès, C. *et al.* Noise pollution on coral reefs?—A yet underestimated threat to coral reef communities. *Mar. Pollut. Bull.* **165**, 112129 (2021).

2. McCloskey, K. P. *et al.* Assessing and mitigating impacts of motorboat noise on nesting damselfish. *Env. Pollut.* **266**, 115376 (2020).

3. Simpson, S. D. *et al.* Small-boat noise impacts natural settlement behaviour of coral reef fish larvae. *The effects of noise on aquatic life II* 1041–1048 (Springer, New York, 2016).
