# Supplementary material for: Unlocking the soundscape of coral reefs with artificial intelligence: pretrained networks and unsupervised learning win out
Source: PLoS Comput Biol. 2025 Apr 28;21(4):e1013029. doi: 10.1371/journal.pcbi.1013029 (PMC12064026; doi:10.1371/journal.pcbi.1013029)
Supplement: S3 Text — (DOCX) [file pcbi.1013029.s013.docx]

**S3 Text: Interactive UMAP plots**

Once opened, users can hover their cursor over the individual points to reveal temporal metadata about each point and the site of origin. Points are coloured by site. Recordings from similar times periods show a clear pattern of grouping together. Further exploration also reveals other temporal insights. For example, on the French Polynesian plot, the ‘bridges’ between primary clusters for each site are dominated by recordings taken during crepuscular periods where ‘hr of day’ is 5-7am or 17-19pm. In the Indonesian plot, an area of overlap between site A and C is visible during the new moon period at 5pm and 5am respectively, showing the soundscapes converged during this period. Other patterns can likely be revealed using this tool.
